# Integrated Analysis of Microbiome and Metabolome Reveals Disease-Specific Profiles in Inflammatory Bowel Diseases and Intestinal Behçet’s Disease

**DOI:** 10.3390/ijms25126697

**Published:** 2024-06-18

**Authors:** Yehyun Park, Jae Bum Ahn, Da Hye Kim, I Seul Park, Mijeong Son, Ji Hyung Kim, Hyun Woo Ma, Seung Won Kim, Jae Hee Cheon

**Affiliations:** 1 Department of Internal Medicine, Yonsei University College of Medicine, Seoul 03722, Republic of Korea; splendidyh1029@ewha.ac.kr (Y.P.); jaebum89@yuhs.ac (J.B.A.); junfeel@hanmail.net (D.H.K.);; 2 Department of Internal Medicine, Ewha Womans University Seoul Hospital, Seoul 03760, Republic of Korea; 3 Brain Korea 21 PLUS Project for Medical Science, Yonsei University College of Medicine, Seoul 03722, Republic of Korea

**Keywords:** intestinal Behçet’s disease, ulcerative colitis, Crohn’s disease, microbiome, metabolome, multi-omics

## Abstract

The gut microbial and metabolic characteristics of intestinal Behçet’s disease (BD), a condition sharing many clinical similarities with ulcerative colitis (UC) and Crohn’s disease (CD), are largely unexplored. This study investigated the gut microbial and metabolic characteristics of intestinal BD as well as potential biomarkers, comparing them with those in UC, CD, and healthy controls. Colon tissue and stool samples from 100 patients (35 UC, 30 CD, and 35 intestinal BD) and 41 healthy volunteers were analyzed using 16S ribosomal RNA sequencing to assess microbial diversity, taxonomic composition, and functional profiling. Plasma metabolomic analyses were performed using gas chromatography and ultra-performance liquid chromatography-mass spectrometry. Results indicated reduced microbial diversity in CD but not in intestinal BD, with intestinal BD showing fewer changes compared to controls yet distinct taxonomic features from UC, CD, and controls. Common alterations across all diseases included a reduction in beneficial bacteria producing short-chain fatty acids. Intestinal BD-specific changes featured a decreased abundance of Bacteroides fragilis. Metabolomic profiles in intestinal BD were similar to those in CD but distinct from those in UC, displaying significant changes in energy metabolism and genetic information processing. This integrative analysis revealed both shared and unique profiles in intestinal BD compared with UC, CD, and controls, advancing our understanding of the distinctive features of these diseases.

## 1. Introduction

Crohn’s disease (CD) and ulcerative colitis (UC) are together called inflammatory bowel disease (IBD), a chronic inflammatory disorder of the gastrointestinal tract that is related to dysbiosis and altered interactions between the dysbiotic microbiota and the host intestinal immune system [1]. In CD, transmural inflammation can occur throughout the entire gastrointestinal tract, whereas inflammation in UC is confined to the mucosal or submucosal layer of the colon. Behçet’s disease (BD) is a chronic relapsing systemic inflammatory disorder of unknown origin characterized by oral and genital mucosal ulcers, uveitis, skin lesions, and neurological or gastrointestinal manifestations. The prevalence of intestinal involvement in patients with BD has been reported in the range of 2.8% to 50% with remarkable geographic variation, and it is more frequent in East Asia, including Korea and Japan, than in other areas of the world [2,3]. IBD and intestinal BD have many similarities: chronic inflammation in the gastrointestinal tract, extraintestinal manifestations, and chronic fluctuating courses characterized by repeated episodes of relapse and remission. IBD and intestinal BD might be closely related and parts of a spectrum of one disease rather than distinct disease entities. It is postulated that, similar to IBD, the pathogenesis of intestinal BD involves triggering events, such as infection, in genetically predisposed individuals [4]. However, due to its rarity in Western countries, understanding of the pathogenesis of intestinal BD remains significantly less established than that of IBD [5]. Although numerous studies have suggested the involvement of intestinal microbiota in the pathogenesis of IBD, such investigations are largely lacking in the context of intestinal BD.

The development of cultivation-independent methods based on next-generation sequencing has rapidly expanded knowledge about the fundamental role of the intestinal microbiome in the pathogenesis of diseases in the gastrointestinal tract. Previous studies have evaluated the composition of the gut microbiota in IBD patients and confirmed significant differences from healthy individuals [6,7,8,9,10,11,12,13,14]. However, previous studies about specific changes in the intestinal microbiota of IBD patients have reported heterogeneous results, and few data about intestinal BD are available. Furthermore, simply understanding variations in the microbial community structure is insufficient to fully grasp the pathogenesis and characteristics of these diseases. An emerging field of study, multi-omics involves integrating various types of chemical and biological data to offer a comprehensive, functional, and mechanistic understanding of complex biological systems. One of the types of data integrated with marker gene sequencing is metabolite data. Metabolomics is the study of the metabolome, the collective array of metabolites present in a biological sample. Metabolomic data provide important information about molecules such as short-chain fatty acids (SCFAs) and bile acids that are produced or modified by the gut microbiota and affect mucosal protection and immune regulatory functions. Due to the inherent limitations of 16S ribosomal RNA (rRNA) gene sequencing in estimating microbial community function, the integration of metabolomics provides a more comprehensive understanding of both the composition and function of microbial communities. Several studies have identified metabolite differences between the stool [15,16], serum [16,17,18], or mucosa of IBD patients and those of controls. Although fecal metabolites might best reflect the direct metabolic output of the microbiota, blood metabolites offer insight into the subset of those compounds that enter circulation, potentially influencing host metabolism and health. The advent of untargeted metabolomics has expanded comprehension of the blood metabolome and facilitated the detection of distinctive molecules produced by the gut microbiota that are present in circulation and might have biological effects on the host. However, such a multi-omics approach has mainly been conducted within the broader context of IBD, and research distinguishing features between UC and CD is lacking. Specifically, studies are needed to integrate the characteristics of BD, UC, and CD for analysis.

Our aim in this study was to compare the gut microbiomes of patients with intestinal BD, UC, and CD with those of healthy controls and to identify alterations in plasma metabolites. Through this study, we sought to discover microbial and metabolomic markers that can aid in diagnosing and differentiating UC, CD, and intestinal BD.

## 2. Results

### 2.1. Ulcerative Colitis, Crohn’s Disease, and Intestinal Behçet’s Disease Display Distinct Tissue and Fecal Microbiota Compositions

**Figure 1:**
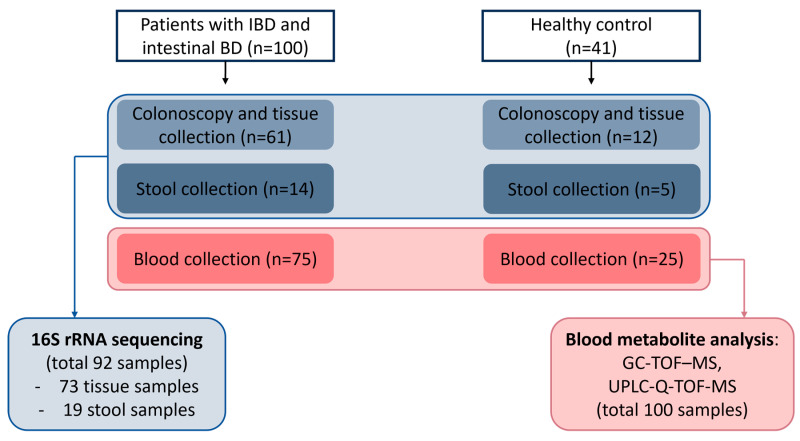
Flow diagram of the study. In total, 192 samples, comprising tissue, stool, and blood, were collected from 141 subjects (35 UC, 30 CD, 35 intestinal BD, and 41 healthy volunteers) and analyzed. IBD: inflammatory bowel disease, BD: Behçet’s disease, rRNA: ribosomal ribonucleic acid, GC-TOF-MS: gas chromatography time-of-flight mass spectrometry, UPLC-Q-TOF-MS: ultra-performance liquid chromatography-quadrupole/time-of-flight mass spectrometry.

**Figure 2:**
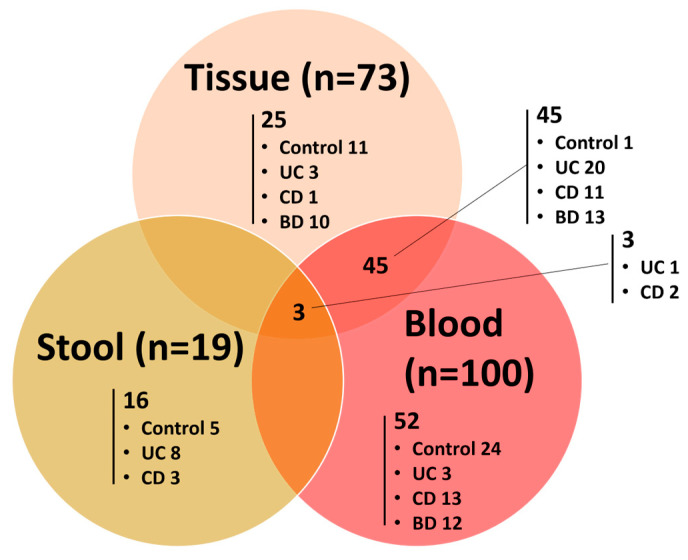
Venn diagram of samples collected from 141 patients or controls according to sample type. UC: ulcerative colitis, CD: Crohn’s disease, BD: Behçet’s disease.

We enrolled 100 patients (35 UC, 30 CD, and 35 intestinal BD) and 41 healthy volunteers in this study. We aimed to collect tissue, stool, and blood samples from each patient, but to ensure an adequate number of subjects for analysis, we also included those who could only provide one or two types of samples. Overall, 192 samples from 141 subjects were analyzed. We conducted 16S rRNA sequencing on 73 tissue samples (12 control, 24 UC, 14 CD, and 23 intestinal BD) and 19 stool samples (5 control, 9 UC, and 5 CD), and we performed metabolite analyses on 100 blood samples (25 control, 24 UC, 26 CD, and 25 intestinal BD) (Figure 1). The sample status of the 141 patients and controls is visualized in Figure 2. Clinical information for the patients and controls who contributed each sample type is shown in Table 1.

**Figure 3:**
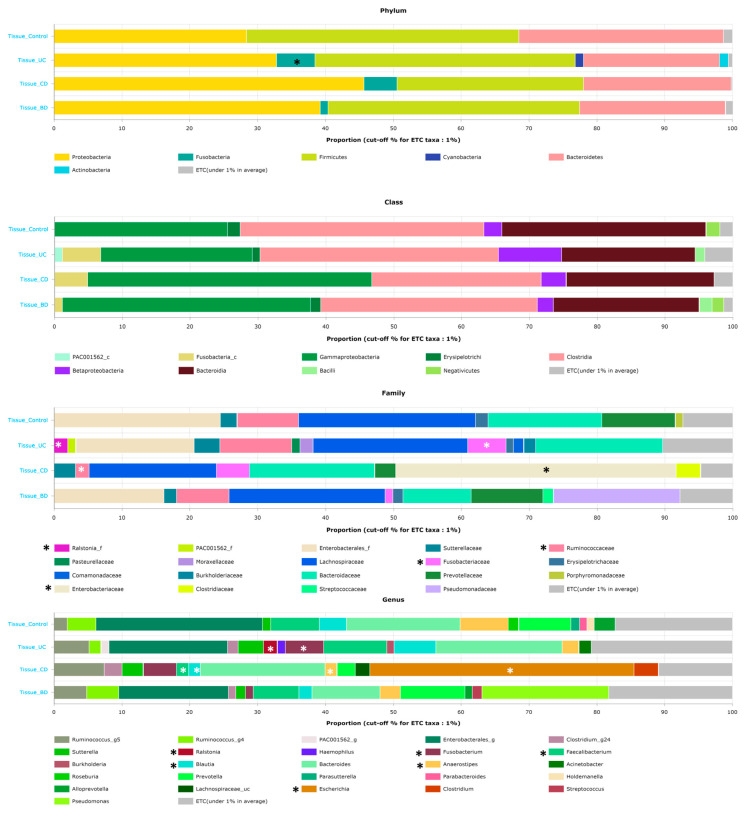
Stacked bar chart of the microbial composition in colon tissue. The microbial analyses of fecal samples from the control and IBD participants showed different patterns. At the phylum level, IBD showed decreased Firmicutes and Actinobacteria and increased Proteobacteria and Bacteroidetes compared with the control (Supplementary Figure S1). The asterisk (*) indicates microbiota that show significant differences when compared to the control.

To investigate differences in the microbial composition in IBD and intestinal BD, the relative abundance of multiple taxa was compared between the control and IBD or intestinal BD samples. At the phylum level, IBD and intestinal BD had an increased tendency for Proteobacteria and Fusobacteria and a decreased tendency for Bacteroidetes compared with the control (Figure 3). Fusobacteria in UC were significantly increased compared with the control (*p* < 0.05). UC had increased abundance of the order *Fusobacteriales*, families *Fusobacteriaceae* and *Burkholderiaceae*, and genera *Ralstonia* and *Fusobacterium,* and decreased abundance of the genus *Roseburia*. CD showed increased abundance of the order *Enterobacterales*, family *Enterobacteriaceae*, and genus *Escherichia* and decreased abundance of the families *Ruminococcaceae* and *Coriobacteriaceae* and genera *Blautia*, *Anaerostipes*, *Faecalibacterium*, and *Roseburia*. In intestinal BD, decreased abundance of the family *Bacteroidaceae* and genera *Bacteroides*, *Acinetobacter*, and *Subdoligranulum* was noted, but those changes were only significant compared with the control; no significant difference compared with IBD was found.

**Figure 4:**
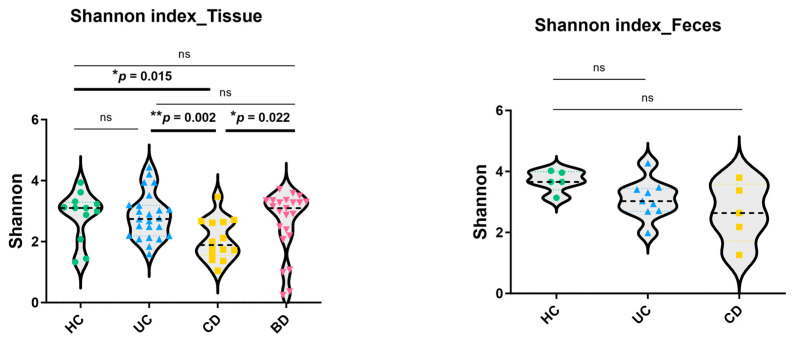
Microbial α-diversity index from tissue and fecal samples. Microbial richness and evenness were evaluated by the Shannon index. Each small shape represents an individual patient. In the tissue samples, CD had a significant decrease in α-diversity compared with the control, UC, and intestinal BD. HC: healthy control, CD: Crohn’s disease, UC: ulcerative colitis, BD: Behçet’s disease, ns: not significant.

**Figure 5:**
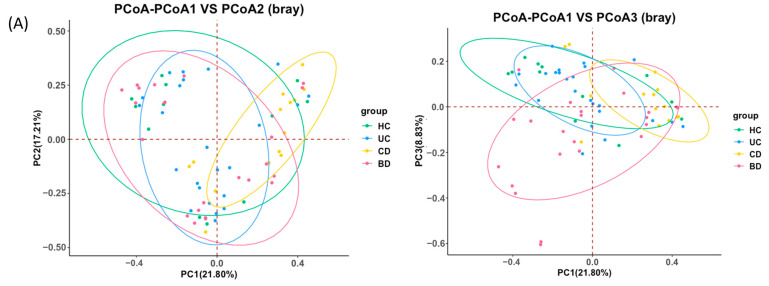

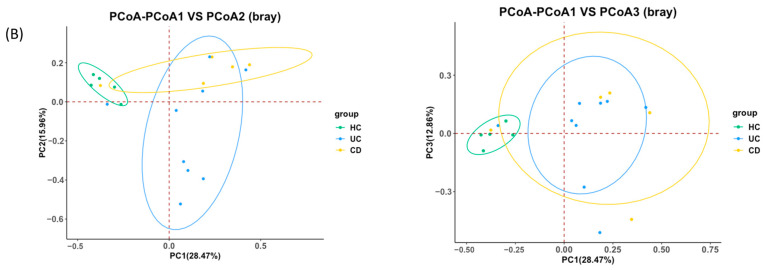
Principal coordinate analysis (PCoA) plots of tissue samples (**A**) and stool samples (**B**). Beta-diversity was analyzed by the Bray-Curtis method. (**A**) PCoA plot for tissue samples shows clustering according to groups and significantly different microbial compositions between the control and CD, the UC and CD, the UC and intestinal BD, and the CD and intestinal BD samples (all *p* < 0.05). (**B**) PCoA plot for fecal samples shows greater separation between IBD and the control and significantly different microbial composition between the control and UC and the control and CD samples (all *p* < 0.05). HC: healthy control, UC: ulcerative colitis, CD: Crohn’s disease, BD: Behçet’s disease.

Microbial richness and evenness were evaluated by the Shannon index, and the tissue sample analysis showed a significant decrease in α-diversity in CD compared with control, UC, and intestinal BD samples (Figure 4). In the fecal sample analysis, the Shannon index was not different in UC and CD, but the number of OTUs and phylogenetic diversity decreased in UC compared with the control (Supplementary Figure S2). Beta-diversity analysis was performed by the Bray-Curtis method. The PCoA plot from the tissue samples showed clustering according to groups and significantly different microbial compositions between the control and CD (PERMANOVA *p* value = 0.011), the UC and CD (PERMANOVA *p* value = 0.004), the UC and intestinal BD (PERMANOVA *p* value = 0.01), and the CD and intestinal BD (PERMANOVA *p* value = 0.002) (Figure 5A) samples. The PCoA plot from the fecal samples showed greater separation between IBD and the control and significantly different microbial composition between the control and UC (PERMANOVA *p* value = 0.002) and the control and CD (PERMANOVA *p* value = 0.025) (Figure 5B).

### 2.2. Intestinal BD Displays Distinctive Microbiota in Both Tissue and Feces, Unlike Other IBDs

**Figure 6:**
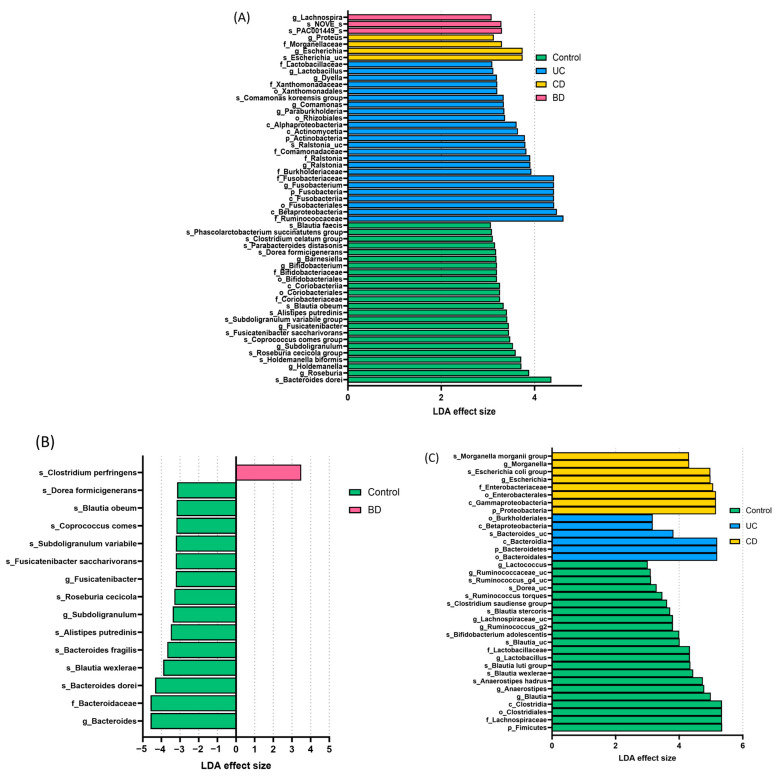
Taxonomic biomarkers analyzed by LEfSe of tissue samples (**A**,**B**) and fecal samples (**C**). Taxa with an LDA effect size > 3 and *p* < 0.05 were visualized. (**A**) LEfSe analysis of tissue samples demonstrated significantly different abundances of specific taxa among the control, UC, CD, and intestinal BD samples. (**B**) LEfSe analysis performed separately for intestinal BD vs. the control showed a decreased abundance of butyrate-producing bacteria in intestinal BD. (**C**) LEfSe analysis of fecal samples also demonstrated significantly different abundances of specific taxa among the control, UC, and CD samples. LDA: linear discriminant analysis, LEfSe: LDA effect size, UC: ulcerative colitis, CD: Crohn’s disease, BD: Behçet’s disease.

The LEfSe analysis of tissue samples demonstrated significantly different abundances of specific taxa in the control, UC, CD, and intestinal BD samples. Taxa with an LDA effect size > 3 and *p* < 0.05 are visualized in Figure 6A. In the control group, the class Coriobacteriia, orders *Coriobacteriales* and *Bifidobacteriales*, families *Coriobacteriaceae* and *Bifidobacteriaceae*, and genera *Roseburia*, *Holdemanella*, *Subdoligranulum*, *Fusicatenibacter*, *Bifidobacterium*, and *Barnesiella* were more abundant than in the UC, CD, or intestinal BD samples, making them potential biomarkers for discriminating healthy status versus IBD or intestinal BD. In UC, the phylum Fusobacteria; classes Alphaproteobacteria, Actinomycetia, Fusobacteriia, and Betaproteobacteria; orders *Xanthomonadales*, *Rhizobiales*, and *Fusobacteriales*; families *Lactobacillaceae*, *Xanthomonadaceae*, *Comamonadaceae*, *Ralstonia*, *Burkholderiaceae*, *Fusobacteriaceae*, and *Ruminococcaceae*; and genera *Lactobacillus*, *Dyella*, *Comamonas*, *Paraburkholderia*, *Ralstonia*, and *Fusobacterium* were differentially abundant compared with the control, CD, and intestinal BD samples. In CD, the family *Morganellaceae* and genera *Proteus* and *Escherichia* were more abundant than in the control, UC, or intestinal BD samples. In intestinal BD, the genus *Lachnospira* was the only taxon with a valid name that was differentially abundant compared with the control and IBD samples in the LEfSe analysis.

**Figure 7:**
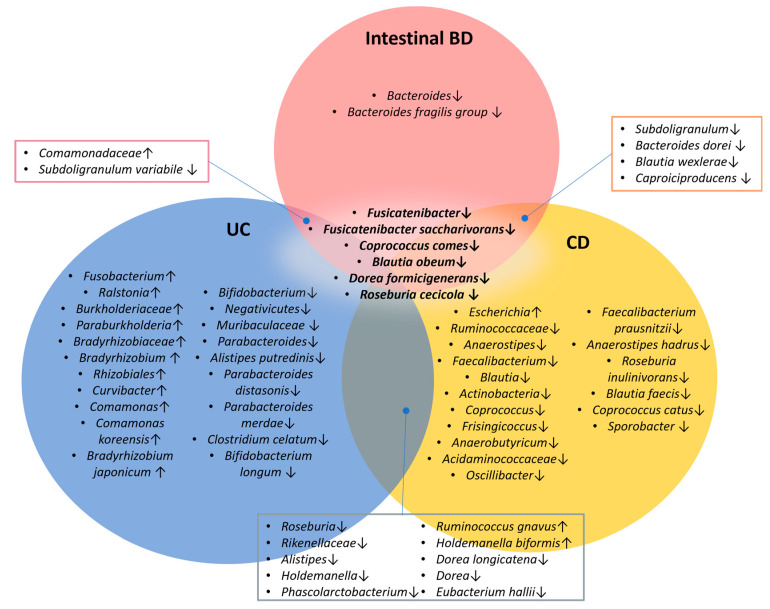
Microbial taxonomic biomarkers of IBD and intestinal BD. The downward arrow indicates a decrease in the respective microbiota, while the upward arrow indicates an increase. The genus *Fusicatenibacter* and species *Fusicatenibacter saccharivorans*, *Coprococcus comes*, *Blautia obeum*, *Dorea formicigenerans*, and *Roseburia cecicola* consistently exhibited decreased abundance, indicating their ‘protective’ role in UC, CD, and intestinal BD. Intestinal BD displayed fewer significant changes, mainly characterized by a decrease in the abundance of several taxa. IBD: inflammatory bowel disease, UC: ulcerative colitis, CD: Crohn’s disease, BD: Behçet’s disease.

The LEfSe analysis performed separately to compare intestinal BD with the control showed a decreased abundance of butyrate-producing bacteria such as *Dorea formicigenerans*, *Subdoligranulum variabile*, *Roseburia cecicola*, *Coprococcus comes*, and *Caproiciproducens* (Figure 6B). The LEfSe analysis of fecal samples also demonstrated that the control, UC, and CD tissue samples had significantly different abundances of specific taxa (Figure 6C). However, simultaneous LEfSe analysis for all four groups might not effectively capture taxa that are consistently increased or decreased across groups because it tends to highlight features unique to a single group. To identify taxa commonly increased or decreased in UC, CD, and intestinal BD, we conducted separate LEfSe analyses for control vs. UC, control vs. CD, and control vs. intestinal BD. Taxa that showed differences (LDA > 2.5, *p* < 0.05) in those analyses were further assessed using the Mann-Whitney *U* test, and only those taxa with significant taxonomic composition changes were selected as microbial biomarkers and visualized in a Venn diagram (Figure 7). The genus *Fusicatenibacter* and species *Fusicatenibacter saccharivorans*, *Coprococcus comes*, *Blautia obeum*, *Dorea formicigenerans*, and *Roseburia cecicola* consistently exhibited decreased abundance, indicating their ‘protective’ role in UC, CD, and intestinal BD. UC exhibited the most dynamic changes, with many increased and decreased taxa, whereas CD primarily had a decrease in the abundance of multiple taxa. Intestinal BD, on the other hand, displayed fewer significant changes from the control, and those changes were mainly characterized by a decrease in the abundance of *Subdoligranulum variabile* and *Blautia wexlerae*. As a specific alteration unique to intestinal BD, a decrease in the genus *Bacteroides*, particularly the species *Bacteroides fragilis*, was identified.

### 2.3. Inflammatory Bowel Diseases Exhibit Pronounced Functional Profiling of the Microbiome According to Subtype

**Figure 8:**
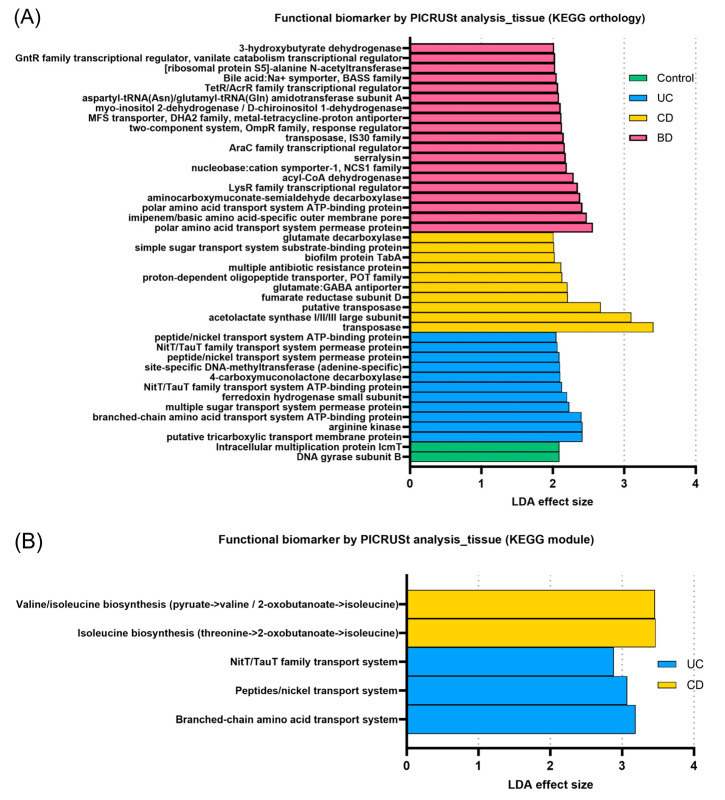
Predictive functional profiling based on PICRUSt analysis of tissue samples. (**A**) KEGG orthology. (**B**) KEGG module. The PICRUSt analysis revealed multiple enhanced gene allocations for each group. Intestinal BD exhibited pronounced enhancements in functions related to drug resistance, signaling and cellular processes, and metabolic pathways. PICRUSt: phylogenetic investigation of communities by reconstruction of unobserved states, KEGG: Kyoto Encyclopedia of Genes and Genomes, UC: ulcerative colitis, CD: Crohn’s disease, BD: Behçet’s disease.

Based on 16S rRNA gene sequencing data from the tissue samples, we performed predictive functional profiling of the microbiomes using PICRUSt analysis annotated to KEGG orthology (KOs). In total, gene allocation for 42 KOs (2 in control, 11 in UC, 10 in CD, and 19 in intestinal BD) was differentially enhanced between groups with significance (LDA > 2.0, *p* < 0.05, FDR-adjusted *p* < 0.1) (Figure 8). The PICRUSt analysis of the microbiomes of fecal samples showed no KOs, pathways, or modules with an FDR-adjusted *p* < 0.1. Intestinal BD exhibited pronounced functional changes, including orthology related to drug resistance and signaling, cellular processes, and metabolic pathways.

### 2.4. Intestinal Behçet’s Disease Metabolomic Profiles Share Similarities with Crohn’s Disease and Diverge from Ulcerative Colitis

**Figure 9:**
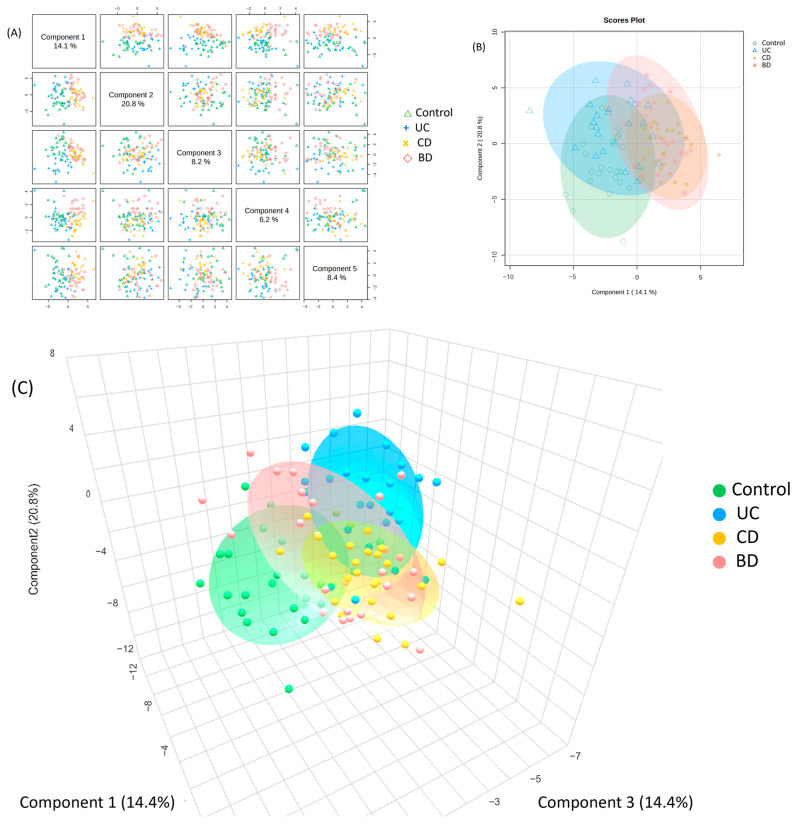
Partial least squares discriminant analysis (PLS-DA) of plasma metabolites by GC-TOF-MS analysis. (**A**) Using five components, we can identify combinations of two components that best explain the differences between groups. Components 1 and 2 have high explanatory power and effectively illustrate the differences between groups. (**B**) PLS score 2D plot generated using components 1 and 2. The plot demonstrates the segregation of three groups: control, UC, and a combined group of CD and intestinal BD. (**C**) PLS score 3D plot using components 1, 2, and 3. GC-TOF-MS: gas chromatography time-of-flight mass spectrometry, UC: ulcerative colitis, CD: Crohn’s disease, BD: Behçet’s disease.

**Figure 10:**
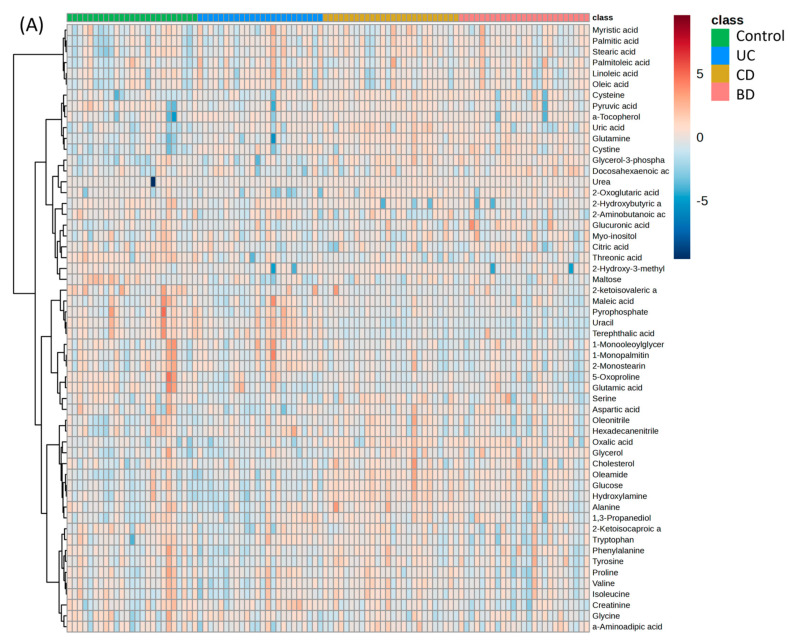

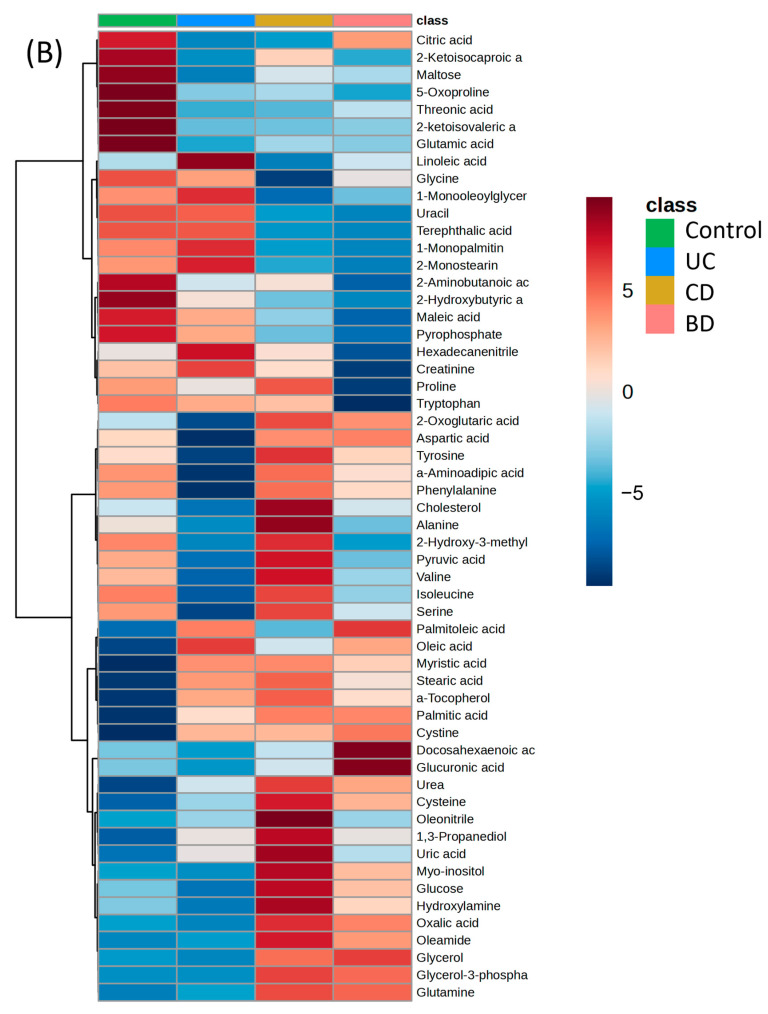
Heatmap of whole plasma metabolite profiles in individual samples (**A**) and by group (**B**). Overall, the metabolite profiles showed similarity between the control and UC and between CD and intestinal BD. UC: ulcerative colitis, CD: Crohn’s disease, BD: Behçet’s disease.

A GC-TOF-MS metabolomic analysis of 100 individual samples (25 control, 24 UC, 26 CD, and 25 intestinal BD) was performed. In the PCA score plots, the control and UC formed one cluster, and CD and intestinal BD constituted another distinct cluster. The PLS-DA score plot derived from the value of the PCA model demonstrates the segregation of three groups: control, UC, and a combined group of CD and intestinal BD (Figure 9A,B). Despite the significant difference among the three clusters (*p* < 0.05), reproducibility and predictability were low (R^2^ = 0.43, Q^2^  =  0.30), and a clear separation between CD and intestinal BD was not achieved. Consequently, a 3D PLS-DA score plot was constructed, and it exhibited distinct separation among all four groups (Figure 9C). The PLS-DA cross-validation data showed cumulative values of R^2^ = 0.61 and Q^2^ = 0.52, where R^2^ indicates the variation shown by all five components in the model and Q^2^ shows the predictability when all five components are considered (Supplementary Figure S3). These score plots and values indicate good clustering and demonstrate a good distinction among the four groups. Subsequently, we used this model to identify the metabolites that contribute to group differentiation. The whole metabolomics profiles are shown as a heatmap (Figure 10). The Spearman rank correlation analysis between metabolites is shown in a correlation heatmap (Supplementary Figure S4). Metabolites within the same categories, such as fatty acids or amino acids, exhibited positive correlations within each category.

**Figure 11:**
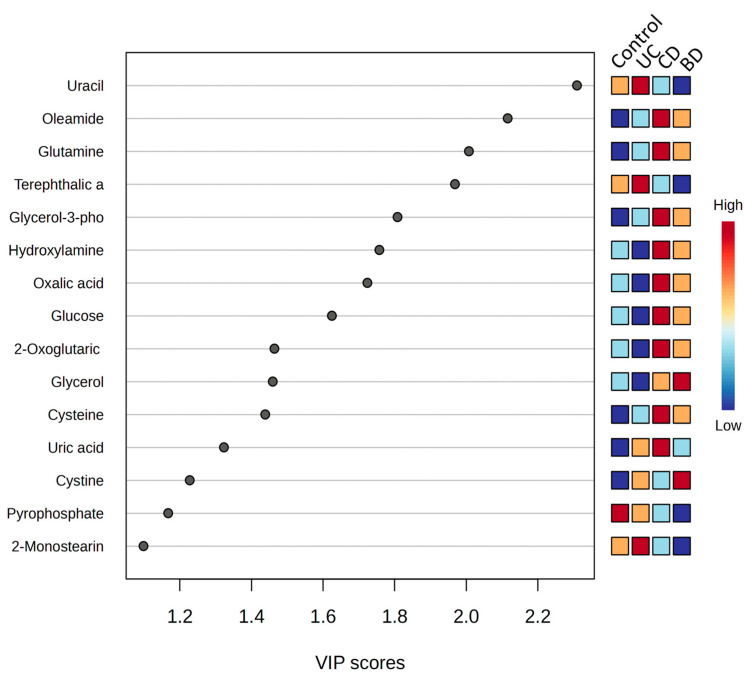
Plasma partial least squares discriminant analysis (PLS-DA) variable importance in a projection (VIP) plot. The key metabolites that contributed most to the separation among controls, UC, CD, and intestinal BD samples are shown in a PLS-DA VIP plot ranked by importance. UC: ulcerative colitis, CD: Crohn’s disease, BD: Behçet’s disease.

The key metabolites that contributed most to the separation between controls, UC, CD, and intestinal BD, are shown in a PLS-DA VIP plot ranked by importance (Figure 11). The VIP scores rank the overall contribution of each variable to the PLS-DA model. Uracil was shown to be a top metabolite in the discriminant analysis, with a higher level in UC, lower levels in CD and intestinal BD, and an intermediate level in the control. The other top metabolites, oleamide, glutamine, glycerol-3-phosphate, hydroxylamine, oxalic acid, glucose, 2-oxoglutaric acid, glycerol, and cysteine, had similar patterns of decrease in UC and increase in CD and intestinal BD compared with the control.

**Figure 12:**
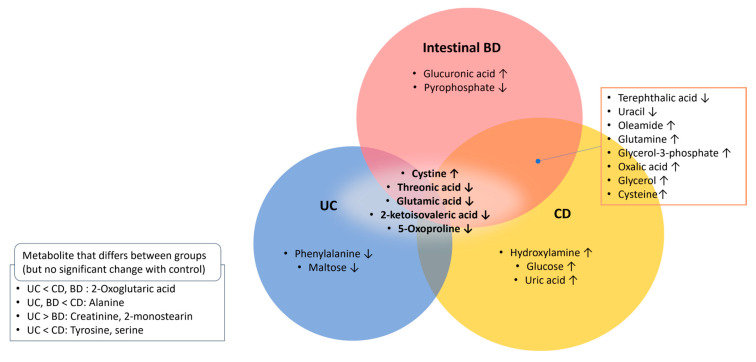
Potential metabolomic biomarkers of IBD and intestinal BD. In total, 26 metabolites with a VIP > 1.0, ANOVA *p* < 0.05, and FDR-adjusted *p* < 0.1 (20 different from control, 6 different among UC, CD, and intestinal BD) were noted. IBD: inflammatory bowel disease, UC: ulcerative colitis, CD: Crohn’s disease, BD: Behçet’s disease, VIP: variable importance in projection, ANOVA: analysis of variance, FDR: false discovery rate. The downward arrow indicates a decrease in the respective microbiota, while the upward arrow indicates an increase.

Among the 56 metabolites on the heatmap, 26 with a VIP >1.0 in the selected PLS-DA model, *p* < 0.05, and FDR *p* < 0.1 by ANOVA were identified and selected as metabolites that differ among the control, UC, CD, and intestinal BD samples. These potential metabolomic biomarkers of IBD and intestinal BD are summarized as a Venn diagram (Figure 12, Supplementary Table S1). Common changes were observed among the three groups in comparison to the control group: an increase in cystine and a decrease in threonic acid, glutamic acid, 2-ketoisovaleic acid, and 5-oxoproline. Substantial overlap was observed between intestinal BD and CD, characterized by a decrease in terephthalic acid and uracil and an increase in oleamide, glutamine, glycerol, glycerol-3-phosphate, oxalic acid, and cysteine. UC exhibited a distinct metabolite profile compared with the other two conditions. UC had the least pronounced metabolite changes compared with the control, with noted decreases in phenylalanine and maltose. In CD, a distinct increase in hydroxylamine, glucose, and uric acid was noted compared with the control and other groups. In intestinal BD, a distinct increase in glucuronic acid and a decrease in pyrophosphate were notably observed.

UPLC-Q-TOF-MS metabolomic analyses of 100 individual blood samples (25 control, 24 UC, 26 CD, and 25 intestinal BD) were also performed, and 18 lysophospholipids, including lysophosphatidylcholine and lysophosphatidylethanolamine (lysoPE), were identified. However, those lysophospholipids did not show significant differentiation between groups in either the PCA score plots or the PLS-DA score plots. The entire metabolomics profiles from the UPLC-Q-TOF-MS analyses are shown as a heatmap (Supplementary Figure S5). Similar to the GC-TOF-MS analysis, the control and UC appeared similar, and intestinal BD and CD appeared similar. However, aside from an increase in lysoPE in CD, there were no specific trends.

### 2.5. Functional Aspects of Metabolomic Biomarkers in Inflammatory Bowel Diseases and Intestinal Behçet’s Disease Differ from Those in Healthy Controls

**Figure 13:**
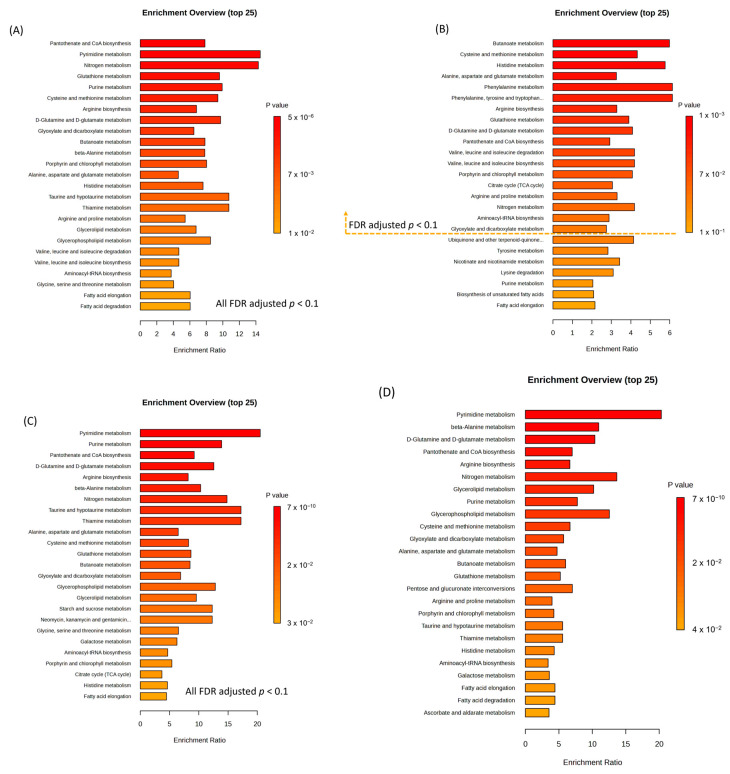
Quantitative enrichment analysis (QEA) in IBD and intestinal BD (**A**), UC (**B**), CD (**C**), and intestinal BD (**D**) compared with the control and mapped to KEGG pathways (FDR-adjusted *p* < 0.1). IBD: inflammatory bowel disease, UC: ulcerative colitis, CD: Crohn’s disease, BD: Behçet’s disease, KEGG: Kyoto Encyclopedia of Genes and Genomes, FDR: false discovery rate.

To identify biologically meaningful patterns in the metabolomics data, QEA was performed for all diseases collectively (control vs. IBD and intestinal BD) and separately for UC, CD, and intestinal BD. When comparing the control with inflammatory diseases of the bowel (UC, CD, and intestinal BD), 27 pathways, including nitrogen metabolism, nucleotide metabolism, amino acid metabolism, lipid metabolism, carbohydrate metabolism, metabolism of cofactors and vitamins, and genetic information processing, were enhanced (Supplementary Table S2, Figure 13A). In UC, the metabolic pathway analysis revealed 18 pathways that were significantly enriched compared with controls, and 11 of them related to amino acid metabolism (Figure 13B). In CD, 28 enriched metabolic pathways were identified (Figure 13C). In intestinal BD, 28 enriched pathways were identified: 11 related to amino acid metabolism, 4 to lipid metabolism, 6 to carbohydrate metabolism, 3 to cofactor and vitamin metabolism, 1 to energy metabolism, 2 to nucleotide metabolism, and 1 to genetic information processing (Supplementary Table S3, Figure 13D).

### 2.6. Integration of Microbial and Metabolomic Biomarkers Showed Correlations between Functional Aspects of Microbiota and Metabolites

**Figure 14:**
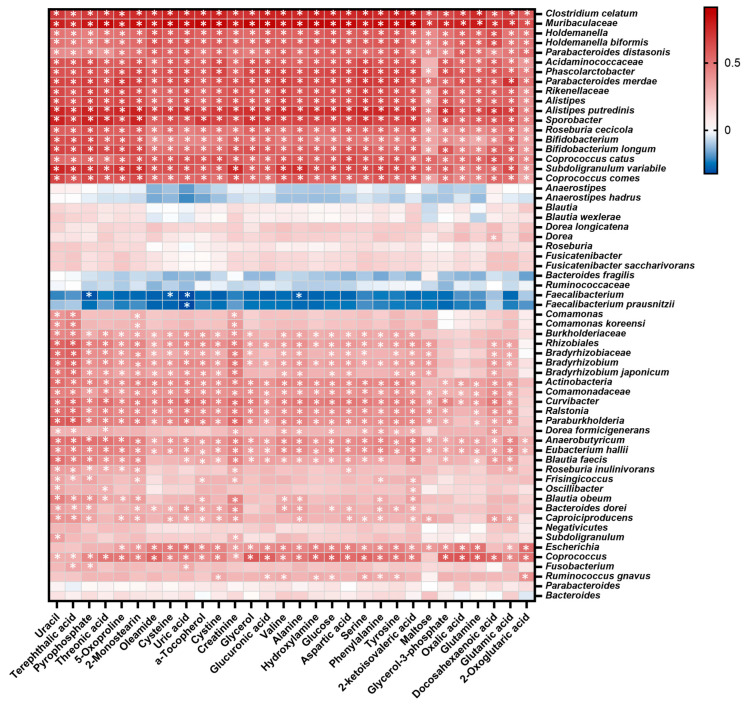
Correlation heatmap of potential microbial and metabolomic biomarkers in UC, CD, and intestinal BD. The Spearman rank correlation analysis between potential microbial and metabolomic biomarkers is shown in the correlation heatmap. The microbial taxa that mainly decreased in UC correlate positively with most of the metabolomic biomarkers, whereas the microbial taxa that mainly decreased in CD and intestinal BD correlate negatively with most of the metabolomic biomarkers. The cells marked with asterisks (*) indicate *p* < 0.05 in the Spearman correlation analysis. UC: ulcerative colitis, CD: Crohn’s disease, BD: Behçet’s disease.

The Spearman rank correlation analysis between potential microbial and metabolomic biomarkers is shown as a correlation heatmap (Figure 14). The microbial taxa that mainly decreased in UC correlated positively with most of the metabolomic biomarkers, which might be associated with the overall decrease in metabolites in UC. On the other hand, the microbial taxa that mainly decreased in CD and intestinal BD correlated negatively with most of the metabolomic biomarkers, leading to an increase in metabolites in CD and intestinal BD.

When we compared the enriched pathways from the PICRUSt microbial functional analysis with the metabolite QEA, we observed a consistent pattern in which the microbial functions showed good alignment with the enriched pathways identified by QEA. In UC, the microbial functions of arginine kinase and branched-chain amino acid transport system ATP-binding protein were increased, corresponding to enriched metabolite pathways for arginine and proline metabolism and valine, leucine, and isoleucine degradation. In CD, the microbial functions of acetolactate synthase I/II/III large subunit, fumarate reductase subunit D, and glutamate decarboxylase were increased, corresponding to enriched metabolite pathways for pantothenate and CoA biosynthesis, butanoate metabolism, citrate cycle, beta-alanine metabolism, taurine and hypotaurine metabolism, and alanine, aspartate, and glutamate metabolism. In intestinal BD, the enriched metabolic pathways that correlated with the PICRUSt results were pyrimidine metabolism; purine metabolism; alanine, aspartate, and glutamate metabolism; butanoate metabolism; arginine and proline metabolism; histidine metabolism; aminoacyl-tRNA biosynthesis; fatty acid degradation; and inositol phosphate metabolism.

## 3. Discussion

This integrative analysis of microbiomes and metabolomes in IBD and intestinal BD identified both common and disease-specific profiles for UC, CD, and intestinal BD. Common changes across all three conditions included a decrease in beneficial bacteria producing SCFAs, such as *Fusicatenibacter saccharivorans*, *Coprococcus comes*, *Blautia obeum*, *Dorea formicigenerans*, and *Roseburia cecicola*. Additionally, reductions in genera such as *Subdoligranulum* and *Roseburia*, previously mentioned in fecal sample studies of systemic BD patients [19], were also observed in intestinal BD in this study and represented shared characteristics between BD and IBD. However, intestinal BD exhibited distinctive features, such as a specific decrease in the genus *Bacteroides*, particularly *Bacteroides fragilis*. The metabolomic profile of intestinal BD was most similar to CD and distinct from both controls and UC. However, UC, CD, and intestinal BD each exhibited distinct metabolomic profiles. Combining microbiome and metabolome results, UC displayed the most dynamic taxonomic changes in the microbiome and the fewest microbial functional alterations and metabolomic changes. In contrast, intestinal BD, which had less substantial taxonomic changes than UC or CD, exhibited pronounced functional changes and metabolite alterations. This difference is likely because UC is characterized by inflammation mostly localized to the mucosa, resulting in a diverse range of changes in the mucosa-associated microbiota as both its cause and consequence. On the other hand, intestinal BD and CD exhibit a more systemic disease pattern, which could be a reason for the greater number of plasma metabolite changes associated with systemic inflammation and the host immune response. By identifying common microbiota and metabolites in intestinal BD, UC, and CD, this research helps elucidate a shared mechanism driving chronic intestinal inflammation and serves as a starting point for exploring the pathogenesis related to intestinal BD’s microbiota. Clinically, this information could assist in differential diagnosis when intestinal BD and CD show similar presentations, potentially utilizing microbial biomarkers such as *Bacteroides fragilis* or other CD-specific microbial changes.

Previous studies have shown significant differences in the gut microbiota of IBD patients compared to healthy individuals [6,7,8,9,10,11,12]. Active IBD is associated with an increased abundance of the phylum Proteobacteria, the genera *Fusobacterium*, *Enterococcus*, and *Streptococcus*, and the species *Escherichia coli* and *Ruminococcus gnavus.* Conversely, IBD is linked to the loss of beneficial taxa such as *Faecalibacterium prausnitzii*, *Christensenellaceae*, *Roseburia*, *Bifidobacterium longum*, *Coprococcus*, *Blautia*, and other butyrate-producing bacteria [6,20,21,22]. However, results varied, and few studies compared microbiota differences between UC and CD [6,22,23]. The microbial changes in UC and CD identified in this study align with previous research on IBD [6,7,8,9,10,11,12,20,21,22]. When considering intestinal BD with IBD, we identified shared protective taxa with decreased abundance in all three conditions. These taxa have frequently been described as decreasing in UC and CD in prior studies, except for *Fusicatenibacter saccharivorans*, which has been less frequently described in IBD. Belonging to Clostridium subcluster XIVa, *F. saccharivorans* was initially isolated and cultured from healthy human feces in 2013 [24]. It produces SCFAs such as lactic acid, acetic acid, and succinic acid and is reported to be decreased in UC and CD [25,26,27]. *F. saccharivorans* plays an anti-inflammatory role by inducing IL-10 and preventing murine acute colitis [25].

Research on the gut microbiome in BD is scant, especially in intestinal BD. Previous studies of BD focused on the oral mucosa or saliva microbiomes of systemic BD patients. Although some studies have investigated the fecal microbiota, none have analyzed the microbiome of colon tissue affected by intestinal BD. In the first study that confirmed gut dysbiosis in BD through stool samples in 2015 [19], 22 BD patients (with an unconfirmed precise ratio of those diagnosed with intestinal BD, only 32% described as having gastrointestinal symptoms) exhibited a decrease in the *Roseburia* and *Subdoligranulum* genera, a finding also observed in this study. Another study reported an increase in *Bacteroides uniformis* in the fecal microbiomes of systemic BD patients without intestinal involvement [28]. Among the intestinal BD patients in the present study, 30% of tissue sample donors and 36% of blood sample donors had intestinal BD without systemic BD. Although it is possible that the microbiome characteristics between intestinal BD and systemic BD are similar, colon tissue in intestinal BD might reveal more specific changes in mucosa-associated microbiota. The microbiota that specifically decreased in intestinal BD was *Bacteroides fragilis* in this study. Bacteroidetes is one of the dominant phyla in healthy individuals, and it is known to decrease in patients with IBD. Many *Bacteroides* species can break down complex polysaccharides, releasing simple carbohydrate products that other bacteria can use. Previous studies showed that *Bacteroides* play a crucial role in the ecological networks of the gut microbiota, and their removal can disrupt those networks [29]. Therefore, *Bacteroides* have the potential to act as ‘foundation species’ that help maintain the gut microbial community. Considering this, the decrease in *Bacteroides* in intestinal BD might explain the pronounced functional changes and metabolite alterations found in this condition, even though it does not exhibit microbial taxonomic changes as substantial as those found in UC or CD. Also, some members of the *Bacteroides* genus have demonstrated anti-inflammatory functions [30,31,32]. *Bacteroides fragilis* and its immunomodulatory symbiosis factor, capsular polysaccharide A (PSA) [33], have been extensively studied and shown to prevent colitis in murine models [31,34,35]. The pathogenesis of BD shares features with autoimmune and autoinflammatory diseases, as well as spondyloarthropathies [4]. One suspected triggering infectious agent is herpes simplex virus (HSV)-1, which can lead to an autoimmune response in BD patients due to its high homology with human heat-shock proteins [4,36]. PSA and *Bacteroides fragilis* have potent immunomodulatory activity and protect against diseases such as herpes simplex encephalitis caused by HSV-1 and autoimmune encephalitis triggered by herpes simplex encephalitis. This protection is achieved by stimulating intestinal toll-like receptor 2–positive plasmacytoid dendritic cells and B cells to secrete IL-10, which in turn induces regulatory T cells to produce both IL-10 and IFN-γ. These regulatory mechanisms collectively suppress pathogenic inflammatory monocytes and neutrophils [30]. The immunomodulatory and autoimmune response-suppressing mechanisms associated with *Bacteroides fragilis* could represent a causal link between the decreased abundance of *Bacteroides fragilis* and intestinal BD. Further experimental studies are needed to determine whether the decrease in *Bacteroides fragilis* is actually related to the pathogenesis of intestinal BD.

Numerous studies have reported substantial alterations in the gut metabolite profiles of patients with IBD [11,15,16,37]. Metabolite profiling can also discriminate between different forms of IBD, such as CD and UC [38,39], and further classify patients with CD as having either ileal or colonic inflammation [40]. Previous metabolomic analyses showed a pronounced separation between CD and the control, whereas UC profiles were more heterogeneous [15]. The findings of this study are similar, with UC exhibiting the fewest metabolomic changes. Intestinal BD had metabolomic profiles similar to those of CD and distinct from the controls. Metabolites can be analyzed in multiple sample types, such as blood, urine, stool, and tissue, and each biosample provides different biochemical information. Because blood metabolites can provide information about systemic metabolism that results from crosstalk between the microbiota and the host, we performed a metabolomic analysis of plasma samples. Representative metabolite changes identified in previous studies of IBD using serum and plasma samples include alterations in branched-chain amino acids, increased level of 3-hydroxybutyrate, and decreased levels of glutamine, histidine, tryptophan, and lipids [41,42]. The frequently reported changes in bile acids, or SCFAs, mainly from stool samples, were not detected in this study. This study found that glutamate was commonly decreased in UC, CD, and intestinal BD. Dysbiosis commonly observed in IBD and intestinal BD could contribute to the decreased levels of glutamate, as butyrate-producing commensal bacteria ferment pyruvate to produce butyrate, whereas pathogenic bacteria, such as *Fusobacterium*, use glutamate as a substrate for butyrate production [43]. Additionally, the bacteria that decreased in all three diseases also possessed the enzyme glutamate synthase, which converts glutamine to glutamate. A decrease in these bacteria might contribute to a decrease in glutamate and an increase in glutamine. The unique change in metabolites in intestinal BD was an increase in glucuronic acid. The enrichment analysis showed increased pentose and glucuronate interconversions in intestinal BD, possibly due to decreased bacteria encoding glucuronate isomerase, such as *Bacteroides fragilis* and *Caproiciproducens*. In CD, increases in hydroxylamine and uric acid were identified in this study. Hydroxylamine, a mutagen formed during nitrification and anaerobic ammonium oxidation, is moderately toxic and harmful to humans [44]. Reports on hydroxylamine in IBD are scarce, necessitating further data. Uric acid is the terminal product of purine nucleoside metabolism through xanthine dehydrogenase. An increase in *Bacteroides* and a decrease in *Faecalibacterium*, *Clostridium*, and *Ruminococcus* result in excessive uric acid production in the liver and insufficient uric acid excretion in the kidney and intestine, raising serum uric acid levels [45]. IBD patients have higher uric acid levels than controls, and the serum uric acid-to-creatinine ratio is associated with disease activity in CD [46]. Additionally, phenylalanine was decreased in UC in this study. Although disturbances in phenylalanine levels in fecal and serum samples in IBD have been inconsistently reported, it inhibits TNF-α production and has an anti-inflammatory role [41].

Integrating multi-omics data poses several challenges. Identifying a metabolite that originates from the microbiome can be complex, and pinpointing the specific microorganism responsible for producing or modifying a particular metabolite is even more daunting. Although the mechanistic links among host diseases, microorganisms, and metabolites are becoming clearer, significant questions about disease-associated metabolites remain unanswered, including whether these metabolites originate from bacteria or result from host metabolism, whether they directly affect bacteria or indirectly influence host physiology, and whether they potentially represent a combination of those scenarios. To answer those questions, it is imperative to move beyond merely identifying correlations among various omics data. Additional investigations, such as comparing metabolites with cultured isolates of specific microbiota or using germ-free or specific pathogen-free mouse models, are essential.

This study has several strengths. First, it is the first investigation to examine mucosa-associated microbiome and metabolome changes in intestinal BD, providing a unique perspective. Additionally, it is the first study to analyze intestinal BD alongside IBD, allowing a comprehensive exploration of both commonalities and differences. Although a few studies have examined microbiome changes in systemic BD, they have focused on the fecal microbiome, and none specifically targeted intestinal BD. Compositionally distinct from the luminal microbiota represented by feces [47], the mucosa-associated microbiota interacts more directly with host epithelial and immune cells through pattern recognition receptors and other signals [48].

This study also has several limitations. First, it has a cross-sectional design, and a single sample might not fully capture temporal changes in the intestinal microbiome. The stability of the microbial community over time, rather than the specific taxa present at a single time point, can be a strong predictor of disease activity [49]. Additionally, many other factors, such as diet, lifestyle, and medications, were not controlled in this study, and those factors might have influenced the bacterial composition or metabolite profiles [8]. Because the subjects were not enrolled in a matched manner across groups, the younger age of CD patients compared with the other groups might also have influenced the microbial composition. Also, distinguishing species using 16S rRNA sequencing can be challenging. Furthermore, our untargeted metabolomics detected a relatively small number of metabolites. Additionally, the overall sample size was relatively limited, and the biological samples used for metagenomics analysis differed from those used for metabolite analysis, further limiting our ability to perform a comprehensive microbiome-metabolite interaction analysis. Another limitation is that we included patients who were already receiving treatment, and some changes in the microbiota and metabolites might have been normalized by the treatment, limiting their utility as diagnostic markers. However, the microbial changes observed in IBD in this study are broadly consistent with those reported in previous studies. Moreover, less prominent changes are observed in the mucosal adherent microbiota than in the luminal or fecal microbiota.

## 4. Materials and Methods

### 4.1. Study Subjects

We included patients from the IBD Clinic of Yonsei University College of Medicine, Severance Hospital, Seoul, Korea, who were aged 18 years and older and had UC, CD, or intestinal BD between January 2014 and January 2019. Patients with evidence of active infection or sepsis at the time of enrollment and those who received antibiotics within the previous 3 months were excluded from the study. The diagnoses of UC, CD, and intestinal BD were based on internationally accepted diagnostic criteria [50,51,52]. Each diagnosis involved various factors, including clinical presentation; endoscopic findings or surgical observations; and radiology, histology, or serology. For intestinal BD, only patients who were finally classified as the “definite” or “probable” types were included in this study [52]. A group of healthy volunteers without a current acute illness, renal failure, diabetes, congestive heart failure, or cirrhosis was also enrolled.

The Institutional Review Board of Severance Hospital, Yonsei University, approved this study (IRB approval number: 4-2013-0805). All patients and controls provided written informed consent, and all methods were performed in accordance with the relevant guidelines and regulations.

### 4.2. Clinical Data Collection

Information about demographic factors, disease duration and location, surgery, medical treatment, disease activity, C-reactive protein levels, and albumin levels was collected for each participant. Disease activity was evaluated using the partial Mayo (pMayo) score for UC, Crohn’s disease activity index (CDAI) for CD, and the activity index for intestinal Behçet’s disease (DAIBD) for BD [53]. Disease severity was classified based on clinical scores. Remission was defined as pMayo below 2, CDAI below 150, and DAIBD below 20. Mild disease was defined as pMayo of 2–4, CDAI 150–219, and DAIBD 20–39; moderate disease as pMayo of 5–7, CDAI 220–450, and DAIBD 40–74; and severe disease as pMayo of 8 or higher, CDAI 451 or higher, and DAIBD 75 or higher.

### 4.3. Collecting Tissue, Blood, and Stool Samples

Tissue samples were collected at the time of the colonoscopy. Three mucosal biopsies were retrieved from the ileocecal area using biopsy forceps and immediately snap-frozen in liquid nitrogen. The tissue was stored at −80 °C until further analysis. If there was active inflammation in the ileocecal area, biopsies were performed from non-ulcerated mucosa whenever possible. Stool samples were collected either at the time of colonoscopy (before bowel preparation) or at a visit to the outpatient clinic. Stool samples of 50–100 mg were kept at 4 °C for less than 24 h and then stored at −80 °C until DNA extraction. For patients who consented to blood collection, 10 mL of blood was collected into an EDTA tube following a 9-h fast. The collected blood was transferred to the laboratory immediately, where it was centrifuged at 1500× *g* for 15 min. Each 300 μL aliquot was stored at −80 °C until further analysis.

### 4.4. Microbiome Analysis

#### 4.4.1. DNA Extraction

Genomic DNA was isolated from fecal samples and mucosal biopsy samples using a FastDNA^TM^ SPIN kit for soil (MP Biomedicals, Irvine, CA, USA) according to the manufacturer’s instructions. The extracted DNA was stored at −80 °C until analysis.

#### 4.4.2. PCR Amplification and 16S rRNA Amplicon Sequencing

PCR amplification was performed using barcoded fusion primers targeting the V1 to V3 regions of the 16S rRNA gene and the extracted DNA as a template in a C1000 Touch thermal cycler (Bio-Rad, Hercules, CA, USA) [54,55,56]. The 16S universal primers 27F (5′-GAGTTTGATCMTGGCTCAG-3′) and 518R (5′-WTTACCGCGGCTGCTGG-3′) were used. For samples collected later, fusion primers 341F (5′-CCTACGGGNGGCWGCAG-3′) and 805R (5′-GACTACHVGGGTATCTAATCC) targeting the V3 to V4 regions of the 16S rRNA gene were used [57]. Among 73 tissue samples, 49 (6 control samples, 15 UC samples, 14 CD samples, and 14 BD samples) were sequenced targeting the V1–3 region, and 24 (6 control samples, 9 UC samples, and 9 BD samples) were sequenced targeting the V3–4 region. The PCR product was confirmed using 1% agarose gel electrophoresis and visualized with a Gel Doc system (BioRad, Hercules, CA, USA). Amplified products were purified using a QIAquick PCR purification kit (Qiagen, Valencia, CA, USA) and quantified using a PicoGreen dsDNA assay kit (Invitrogen, Carlsbad, CA, USA). Equimolar concentrations of amplicons purified from different samples were pooled, and short fragments < 500 bp (non-target products) were removed using Ampure beads (Agencourt Bioscience, Beverly, MA, USA). The quality and product size were assessed on a Bioanalyzer 2100 (Agilent, Palo Alto, CA, USA) using a DNA 7500 chip. Mixed amplicons were subjected to emulsion PCR and then sequenced. Pyrosequencing was carried out at ChunLab, Inc. (Seoul, Republic of Korea) using a GS FLX Titanium system (Roche, Branford, CT, USA) and the Illumina MiSeq platform (Illumina, San Diego, CA, USA).

#### 4.4.3. Microbiome Data Analysis

Sequencing data were analyzed according to previous descriptions [54,55,56]. The reads obtained from the samples were categorized using the unique barcodes of each PCR product. The barcode, linker, and primer sequences were removed from the original sequencing reads. The quality of sequencing was checked manually by secondary-structure-aware alignment using the EzEditor program [58]. After eliminating non-specific amplicons, amplicons not assigned to the target taxon, and chimeras in the quality assessment process, the taxonomic classification of each read was analyzed using EzBioCloud [57] with the database version of PKSSU4.0, an up-to-date prokaryotic 16S database. EzBioCloud contains 16S rRNA gene sequences of type strains with valid published names and representative species-level phylotypes of either cultured or uncultured entries with complete hierarchical taxonomic classifications, from the phylum to the species levels. Calculations of alpha- and beta-diversity indices, biomarker discovery using linear discriminant analysis (LDA) effect size (LEfSe), and phylogenetic investigation of communities by reconstruction of unobserved states (PICRUSt) algorithms [59] were performed after normalization based on the 16S rRNA gene copy number variation. For alpha-diversity, we used the numbers of operational taxonomic units (OTUs) for richness, the Simpson method for evenness, and the Shannon index as a combined measure considering both richness and evenness. Beta-diversity was visualized by hierarchical cluster trees using the unweighted pair group method with the arithmetic mean, analyzed by the Bray-Curtis method, and visualized again using a principal coordinate analysis (PCoA) [60,61]. LEfSe was used to identify specific microbiota that were differentially distributed among different samples and might be available as microbial biomarkers. Predictive functional profiling was conducted using PICRUSt and annotated with Kyoto Encyclopedia of Genes and Genomes (KEGG) pathways. Taxonomic data and alpha-diversity were compared between groups using the Mann-Whitney U test. Beta-diversity between groups was compared using permutational multivariate analysis of variance (PERMANOVA), which is a non-parametric multivariate statistical test [62]. A *p* value less than 0.05 was considered statistically significant.

### 4.5. Metabolomic Analysis

#### 4.5.1. Sample Preparation for Metabolomic Analysis

Metabolites were extracted from 200 μL of plasma. One milliliter of methanol containing 10 μL of internal 2-chlorophenylalanine standard (1 mg/mL in water) was added to the plasma samples and then homogenized with a mixer mill at a frequency of 30 Hz for 5 min and a sonicator for 5 min. After homogenization, the suspension was held at 4 °C for 60 min and then centrifuged at 20,000× *g* at 4 °C for 10 min. The supernatant was filtered through a 0.2 μm polytetrafluoroethylene filter and evaporated using a speed vacuum concentrator (Modulspin 31, Biotron, Wonju, Republic of Korea) [63,64,65]. The final concentration of each analyzed sample was 10 mg/mL. The metabolomic analysis by mass spectrometry was carried out at MetaMass, Inc. (Seoul, Republic of Korea).

#### 4.5.2. GC-TOF-MS Analysis

Each 100-μL sample was re-evaporated for derivatization. Dried samples were oximated and silylated for gas chromatography time-of-flight mass spectrometry (GC-TOF-MS) analysis. For metabolite profiling, a GC-TOF-MS analysis was performed using an Agilent 7890A gas chromatography system coupled with an Agilent 7693 autosampler (Agilent Technologies, Palo Alto, CA, USA) and equipped with a Pegasus III TOF MS (LECO Corp., St. Joseph, MI, USA) system. An Rtx-5MS column (30 m × 0.25 mm, 0.25 µm particle size; Restek Corp., Bellefonte, PA, USA) was used with a constant flow (1.5 mL/min) of helium as the carrier gas. Derivatized samples of 1 µL were injected into the GC in splitless mode. The oven temperature was maintained at 75 °C for 2 min, then increased by 15 °C/min to 300 °C and held there as the final temperature for 3 min. The temperatures of the front inlet and transfer line were 250 and 240 °C, respectively. Electron ionization was conducted at −70 eV, and full scanning over the range of 50–800 *m*/*z* was used for mass data collection [63,64,65].

#### 4.5.3. UPLC-Q-TOF-MS Analysis

Dried extracts were re-dissolved in 250 µL of methanol for the ultra-performance liquid chromatography–quadrupole (UPLC-Q) TOF-MS analysis. UPLC was performed on a Waters ACQUITY UPLC™ system (Waters Corp., Milford, MA, USA) equipped with a binary solvent delivery system, a UV detector, and an auto-sampler. Chromatographic separation was performed on a Waters ACQUITY UPLC BEH C18 column (100 mm × 2.1 mm, 1.7 µm particle size; Waters Corp., Milford, MA, USA), and the injection volume was 5 µL. The column temperature was set at 37 °C, and the flow rate was 0.3 mL/min. The mobile phase consisted of 0.1% *v*/*v* formic acid in water (Solvent A) and 0.1% *v*/*v* formic acid in acetonitrile (Solvent B). The total run time was 14 min, including the re-equilibration of the column to the initial conditions. The gradient parameters were set as follows: 5% solvent B was initially maintained for 1 min, followed by a linear increase to 100% solvent B over 9 min, 100% solvent B for 1 min, and a gradual decrease to 5% solvent B over 3 min. For the MS experiments, a Waters Micromass Q-TOF Premier (Micromass MS Technologies, Manchester, UK) was operated in negative ion mode with an *m*/*z* range of 100–1000. The source temperature was set at 100 °C, the collision energy was set at 10 eV, the collision gas flow was 0.3 mL/min, and the desolvation gas was set to 650 L/h at a temperature of 300 °C. The capillary voltage and sample cone voltage were set at 2.5 kV and 50 V, respectively. The V mode was used for the mass spectrometer, and data were collected in centroid mode with a scan accumulation of 0.2 s. Leucine encephalin was used as the reference lock mass (*m*/*z* 554.2615) by independent LockSpray interference [63,64,65].

#### 4.5.4. Data Analysis

The GC-TOF-MS data were acquired, preprocessed, and converted into the NetCDF format (*.cdf) using LECO Chroma TOF™ software (version 4.44, LECO Corp., St. Joseph, MI, USA). The raw data from the UPLC-Q-TOF-MS analysis were acquired and converted into the NetCDF format (*.cdf) by MassLynx software (version 4.1, Waters Corp., Milford, MA, USA) and MassLynx DataBridge software (version 4.1, Waters Corp., Milford, MA, USA) [64,65]. After conversion, peak detection, retention time correction, and alignment were processed using MetAlign software (version 0.12.5, Wageningen Food Safety Research, Wageningen, The Netherlands). The resulting alignment data were exported to a Microsoft Excel file.

The integrative metabolomic data analysis was performed using MetaboAnalyst 5.0 (http://www.metaboanalyst.ca, accessed on 20 August 2023), a web-based comprehensive metabolomics data processing tool [66]. Each variable in the quantitative data was first normalized by the median value and then log-transformed, centered, and scaled to its mean and standard deviation. A hierarchical cluster analysis was performed to identify clustering patterns, and Spearman’s rank correlation coefficients were used to evaluate the correlation between each pair of features. Dendrograms were visualized through heatmaps, on which each colored cell corresponds to a concentration value. To analyze differences between groups, an analysis of variance (ANOVA) was performed on normalized data. To compare differential metabolites among the control, UC, CD, and BD samples and to explain the maximum separation among groups, unsupervised and supervised multivariate regression techniques, principal component analysis (PCA), and partial least squares discriminant analysis (PLS-DA), respectively, were performed. For each model, the optimal number of components was chosen according to the highest prediction accuracy (Q^2^) estimated using the 5-fold cross-validation technique. The discriminative metabolites were selected based on their variable importance in projection (VIP) values. VIP is one of the important measures of PLS-DA and is a weighted sum of squares of the PLS loadings that takes into account the amount of class variation explained by each dimension. Metabolites were ranked according to their VIP scores, and metabolites with VIP scores greater than 1 were considered significant contributors [66]. To identify biologically meaningful patterns based on the metabolomics data, a quantitative enrichment analysis (QEA) was conducted. Data were mapped to the KEGG human metabolic pathway database, which comprises 84 metabolite sets of normal metabolic pathways. QEA is based on the well-established global test [67] of associations between metabolite sets and the outcome. The algorithm uses a generalized linear model to compute a Q-stat for each metabolite set. The Q-stat is the average of the Q values calculated for each single metabolite, and the Q value is the squared covariance between each metabolite and the outcome. Spearman’s rank correlation coefficients were used to analyze correlations between microbiota and metabolites, and the findings were visualized as a heatmap.

### 4.6. Statistical Analysis

Baseline characteristics were analyzed by descriptive statistics. For continuous variables, median, and range are reported. When comparing two groups, the Mann-Whitney *U* test was used. Multiple groups were first compared with the Kruskal-Wallis *H* test, and if *p* < 0.05, then pairwise comparisons with the Mann-Whitney *U* test were used to compare continuous variables. Categorical variables are reported as frequencies with percentages, and they were compared using Pearson’s χ^2^ test or Fisher’s exact test. Correlations were identified by Spearman’s rank correlation coefficients. All results were considered statistically significant when the two-tailed *p* value was <0.05. To control for the false discovery rate (FDR), *p* values were adjusted for multiple comparisons using the Benjamini and Hochberg method [68]. An FDR of 10%, or FDR-adjusted *p* < 0.1, was considered significant for microbial functional biomarker discovery and in the metabolite analysis [17].

Statistical analysis and chart visualization were performed using SPSS version 20.0 (SPSS Inc., Chicago, IL, USA) and GraphPad Prism V.10.0 (GraphPad Software Inc., San Diego, CA, USA).

## 5. Conclusions

In conclusion, this study performed an integrated analysis of the gut microbiome in tissue and stool samples by 16S rRNA sequencing and plasma metabolite profiling with GC-MS-TOF and UPLC-Q-TOF-MS analyses in patients with intestinal BD, UC, or CD and compared those results with healthy controls. The microbial taxonomic profile of intestinal BD showed the least difference when compared to healthy controls, yet it exhibited distinctive features, setting it apart from both UC and CD, and healthy controls. The metabolomic profile of intestinal BD was most similar to CD and distinct from both the controls and UC. Intestinal BD exhibited pronounced functional changes and metabolite alterations without showing microbial taxonomic changes as substantial as those in UC or CD. We identified potential microbial and metabolomic biomarkers that can group the diseases together and others that can distinguish UC, CD, and intestinal BD. Future studies on *Bacteroides fragilis*’ involvement in the pathogenesis of intestinal BD and validation of the microbial biomarkers identified are needed.

## Figures and Tables

**Table 1:** Baseline clinical characteristics of enrolled patients and controls.

	Tissue Microbiome Analysis (n = 73)				*p*	Stool Microbiome Analysis (n = 19)			*p*	Plasma Metabolite Analysis (n = 100)				*p*
Characteristics	Control (n = 12)	UC (n = 24)	CD (n = 14)	BD (n = 23)		Control (n = 5)	UC (n = 9)	CD (n = 5)		Control (n = 25)	UC (n = 24)	CD (n = 26)	BD (n = 25)	
Age, median (range)	47.2 (32.1–74.2)	42.6 (19.5–68.6)	22.7 (18.0–31.5)	46.2 (25.8–76.5)	<0.001	22.0 (20.5–31.2)	44.3 (18.4–56.3)	18.0 (18.0–38.2)	0.05	33.7 (26.0–43.6)	37.9 (18.0–68.6)	23.3 (18.0–42.6)	44.3 (23.3–62.9)	<0.001
Sex, male/female, n (%)	6 (50.0)/6 (50.0)	13 (54.2)/11 (45.8)	11 (78.6)/3 (21.4)	10 (43.5)/13 (56.5)	0.21	3 (60.0)/2 (40.0)	5 (55.6)/4 (44.4)	5 (100)/0	0.21	8 (32.0)/17 (68.0)	15 (62.5)/9 (37.5)	22 (84.6)/4 (15.4)	14 (56.0)/11 (44.0)	<0.01
BMI, kg/m^2^, median (range)	22.3 (15.6–26.8)	21.8 (16.1–28.7)	21.1 (13.9–22.9)	23.0 (16.0–28.0)	0.10	19.1 (18.9–22.8)	20.7 (16.8–23.3)	20.5 (16.3–29.6)	0.87	21.8 (18.1–24.2)	21.8 (16.1–28.7)	20.6 (13.9–31.2)	21.5 (16.5–24.7)	0.53
Bowel resection history, n (%)	0	0	3 (21.4)	1 (4.3)	0.03	0	1 (11.1)	1 (20.0)	1.00	0	0	1 (3.8)	3 (12.0)	0.31
Disease duration, months, median (range)	-	39.6 (24.0–65.6)	20.3 (7.5–28.3)	27.5 (6.8–72.7)	0.20 †	-	2.6 (1.0–46.0)	2.9 (2.0–12.0)	0.95 †	-	32.1 (1.0–155.0)	7.2 (0–128.0)	5.9 (0–110.0)	<0.01 †
Disease location, n (%)	-													
E1 (proctitis)	-	6 (25)	-	-		-	3 (33.3)	-		-	7 (29.2)	-	-	
E2 (left sided)	-	7 (29.2)	-	-		-	4 (44.4)	-		-	5 (20.8)	-	-	
E3 (pancolitis)	-	11 (45.8)	-	-		-	2 (22.2)	-		-	12 (50.0)	-	-	
L1 (ileal)	-	-	0	-		-	-	1 (20)		-	-	4 (15.4)	-	
L2 (colonic)	-	-	1 (7.1)	-		-	-	0		-	-	2 (7.7)	-	
L3 (ileocolic)	-	-	13 (92.9)	-		-	-	4 (80)		-	-	20 (76.9)	-	
With/without systemic BD, n (%)	-	-	-	16 (69.6)/7 (30.4)		-	-	-		-	-	-	16 (64.0)/9 (36.0)	
Disease activity, remission/mild/moderate/severe, n (%)	-	2 (8.3)/10 (41.7)/7 (29.2)/5 (20.8)	7 (50.0)/2 (14.3)/5 (35.7)/0	4 (17.4)/6 (26.1)/7 (30.4)/6 (26.1)	0.05	-	4 (44.4)/4 (44.4)/1 (33.3)/0	1 (20.0)/2 (40.0)/2 (40.0)/0	0.63	-	4 (16.7)/11 (45.8)/4 (16.7)/5 (20.8)	8 (30.8)/10 (38.5)/8 (30.8)/0	6 (24.0)/6 (24.0)/7 (28.0)/6 (24.0)	0.08
Disease activity score, median (range)														
Partial Mayo score	-	4.5 (3.0–6.0)	-	-		-	2.0 (1.0–6.0)	-		-	3.5 (1.0–9.0)	-	-	
CDAI	-	-	148.5 (73.0–226.0)	-		-	-	186.0 (128.0–358.0)		-	-	184.5 (71.0–366.0)	-	
DAIBD	-	-	-	60.0 (22.5–70.0)		-	-	-		-	-	-	40.0 (10.0–150.0)	
Hb, g/dL, median (range)	13.0 (11.7–16.0)	13.5 (9.7–15.2)	12.9 (10.7–15.8)	13.1 (10.2–16.8)	0.63	14.6 (13.2–15.2)	14.1 (12.5–16.5)	12.7 (10.6–16.0)	0.17	12.4 (11.7–13.1)	13.7 (9.7–15.2)	13.9 (10.0–16.9)	12.5 (6.5–15.3)	0.52
ESR, mm/h, median (range)	14.0 (2.0–62.0)	23.5 (2.0–52.0)	37.0 (3.0–99.0)	33.0 (8.0–106.0)	0.07	7.5 (3.0–24.0)	9.0 (2.0–45.0)	47.0 (17.0–99.0)	0.02	-	14.0 (2.0–84.0)	21.0 (7.0–119.0)	37.0 (2.0–120.0)	0.02 †
CRP, mg/L, median (range)	0.4 (0.3–0.5)	2.2 (0.8–3.9)	7.9 (2.3–23.6)	5.6 (1.5–13.9)	0.02	2.3 (0.6–5.5)	1.0 (0.3–25.5)	18.6 (0.7–91.3)	0.10	-	1.0 (0.1–9.2)	6.5 (0.9–66.2)	2.4 (0.3–28.4)	<0.01 †
Albumin, g/dL, median (range)	4.3 (4.2–4.5)	4.2 (4.0–4.5)	4.0 (3.7–4.5)	4.4 (4.2–4.5)	0.14	4.4 (4.1–4.4)	4.4 (4.1–4.8)	3.7 (3.6–4.4)	0.10	4.5 (4.4–4.6)	4.4 (3.5–5.1)	4.1 (3.4–4.8)	4.2 (3.4–5.1)	0.07
Creatinine, mg/dL, median (range)	0.8 (0.5–1.0)	0.7 (0.5–1.1)	0.8 (0.5–1.0)	0.7 (0.5–1.4)	0.62	0.76 (0.62–0.99)	0.78 (0.56–0.94)	0.67 (0.61–0.95)	0.92	0.7 (0.6–0.8)	0.7 (0.5–1.2)	0.7 (0.5–1.1)	0.8 (0.5–1.2)	0.34
ALT, U/L, median (range)	18 (8.0–30.0)	9 (5.0–34.0)	9.5 (6.0–40.0)	20.0 (7.0–40.0)	<0.01	14.0 (12.0–14.0)	11.0 (9.0–20.0)	10.0 (10.0–12.0)	0.71	19 (17.0–21.0)	11.0 (5.0–34.0)	13.0 (5.0–47.0)	14.0 (6.0–58.0)	0.10
Medication, n (%)														
5-ASA	-	24 (100)	12 (85.7)	23 (100)	0.05 †	-	9 (100)	4 (80.0)	0.36 †	-	24 (100)	24 (92.3)	25 (100)	0.33 †
Steroid	-	3 (12.5)	1 (7.1)	10 (43.5)	0.01 †	-	2 (22.2)	1 (20.0)	1.00 †	-	6 (25.0)	6 (23.1)	13 (52.0)	0.05 †
IMM	-	5 (20.8)	8 (57.1)	5 (21.7)	0.04 †	-	0	4 (80)	0.01 †	-	7 (29.2)	15 (57.7)	7 (28.0)	0.05 †
Anti-TNF	-	4 (16.7)	1 (7.1)	0	0.10 †	-	0	0	-	-	1 (4.2)	1 (3.8)	2 (8.0)	0.84 †

UC: ulcerative colitis, CD: Crohn’s disease, BD: intestinal Behçet’s disease, BMI: body mass index, CDAI: Crohn’s disease activity index, DAIBD: disease activity index for intestinal Behçet’s disease, ESR: erythrocyte sedimentation rate, CRP: C-reactive protein, ALT: alanine aminotransferase, 5-ASA: 5-aminosalicilic acid, IMM: immunomodulator, anti-TNF: anti-tumor necrosis factor. † *p* values were calculated between disease groups, excluding the control group.

## Data Availability

The data presented in this study are available on request from the corresponding authors.

## Supplementary Materials

The following supporting information can be downloaded at: https://www.mdpi.com/article/10.3390/ijms25126697/s1.
