# Supplementary material for: Integrated Analysis of Microbiome and Metabolome Reveals Disease-Specific Profiles in Inflammatory Bowel Diseases and Intestinal Behçet’s Disease
Source: Int J Mol Sci. 2024 Jun 18;25(12):6697. doi: 10.3390/ijms25126697 (PMC11203907; doi:10.3390/ijms25126697)
Supplement: Supplementary file 1 [file ijms-25-06697-s001.zip › ijms-3034342-SI.pdf]

Supplementary Table S1. List of significantly changed and differentially expressed between control, UC, and CD

Name	F-value	P value	FDR adjusted *p* value	Tukey's post-hoc comparisons	Max VIP score
Uracil	23.304	<0.001	<0.001	2-0; 3-0; 2-1; 3-1	2.40
Oleamide	16.482	<0.001	<0.001	2-0; 3-0; 2-1; 3-1	2.12
Glutamine	14.065	<0.001	<0.001	2-0; 3-0; 2-1; 3-1	2.01
Terephthalic acid	13.91	<0.001	<0.001	2-0; 3-0; 2-1; 3-1	1.97
Hydroxylamine	13.181	<0.001	<0.001	2-0; 2-1; 3-1; 3-2	1.76
Glycerol-3-phosphate	10.593	<0.001	<0.001	2-0; 3-0; 2-1; 3-1	1.81
Cystine	10.317	<0.001	<0.001	1-0; 2-0; 3-0	1.51
Glucose	9.8479	<0.001	<0.001	2-0; 2-1; 3-1	1.62
Oxalic acid	9.5713	<0.001	<0.001	2-0; 3-0; 2-1; 3-1	1.72
Uric acid	9.5247	<0.001	<0.001	2-0; 2-1; 3-2	1.40
2-Oxoglutaric acid	9.1817	<0.001	<0.001	2-1; 3-1	1.46
Threonic acid	8.0659	<0.001	<0.001	1-0; 2-0; 3-0	1.38
Glycerol	6.6603	<0.001	0.0016873	2-0; 3-0; 2-1; 3-1	1.46
Cysteine	6.5862	<0.001	0.0017107	2-0; 3-0; 2-1	1.44
Glutamic acid	5.9269	<0.001	0.0035111	1-0; 2-0; 3-0	1.19
Glucuronic acid	5.2158	0.0022235	0.0077824	3-0; 3-1	1.11
Alanine	4.7845	0.0037665	0.012407	2-1; 3-2	1.05
2-ketoisovaleric acid	4.5695	0.0049041	0.015257	1-0; 2-0; 3-0	1.17
Phenylalanine	4.4673	0.0055614	0.015677	1-0; 2-1	1.17
Pyrophosphate	4.4619	0.0055988	0.015677	3-0	1.17
5-Oxoproline	4.3706	0.0062657	0.016709	1-0; 2-0; 3-0	1.10

Creatinine	4.2251	0.0074979	0.019086	3-1	1.27
2-Monostearin	3.8419	0.012051	0.027705	3-1	1.15
Maltose	3.821	0.012369	0.027705	1-0	1.08
Tyrosine	3.3493	0.022237	0.046122	2-1	1.09
Serine	3.2671	0.024636	0.049273	2-1	1.01

Among the 56 metabolites, 26 metabolites with VIP score > 1.0 by PLS-DA model with 5 components and $p < 0.05$, FDR adjusted $p < 0.1$ by ANOVA are selected. For Tukey’s post-hoc comparisons, 0 is control, 1 is UC, 2 is CD, and 3 is BD.

Supplementary Table S2. Result from quantitative enrichment analysis of ulcerative colitis, Crohn's disease, and intestinal Behcet's disease compared with control

	Total compound	Hits	Statistic Q	Raw *p*	FDR adjusted *p*
Pantothenate and CoA biosynthesis	19	5	7.91	<0.001	<0.001
Pyrimidine metabolism	39	2	14.66	<0.001	<0.001
Nitrogen metabolism	6	2	14.45	<0.001	<0.001
Glutathione metabolism	28	4	9.68	<0.001	<0.001
Purine metabolism	65	3	10.02	<0.001	<0.001
Cysteine and methionine metabolism	33	4	9.49	<0.001	<0.001
Arginine biosynthesis	14	5	6.88	<0.001	<0.001
D-Glutamine and D-glutamate metabolism	6	3	9.82	<0.001	<0.001
Glyoxylate and dicarboxylate metabolism	32	5	6.56	<0.001	<0.001
Butanoate metabolism	15	2	7.91	<0.001	0.001
beta-Alanine metabolism	21	2	7.90	<0.001	0.001
Porphyrin and chlorophyll metabolism	30	2	8.10	<0.001	0.001
Alanine, aspartate and glutamate metabolism	28	7	4.64	<0.001	0.002
Histidine metabolism	16	2	7.68	<0.001	0.003
Taurine and hypotaurine metabolism	8	1	10.84	<0.001	0.003
Thiamine metabolism	7	1	10.84	<0.001	0.003
Arginine and proline metabolism	38	3	5.49	0.002	0.005
Glycerolipid metabolism	16	2	6.82	0.002	0.007
Glycerophospholipid metabolism	36	1	8.58	0.003	0.008
Valine, leucine and isoleucine degradation	40	4	4.72	0.010	0.024
Valine, leucine and isoleucine biosynthesis	8	4	4.72	0.010	0.024
Aminoacyl-tRNA biosynthesis	48	12	3.78	0.010	0.024
Glycine, serine and threonine metabolism	33	3	4.08	0.012	0.025
Fatty acid elongation	38	1	6.09	0.013	0.027
Fatty acid degradation	39	1	6.09	0.013	0.027
Fatty acid biosynthesis	47	2	4.17	0.034	0.066
Biosynthesis of unsaturated fatty acids	36	4	3.56	0.049	0.090

Supplementary Table S3. Result from quantitative enrichment analysis of intestinal Behcet's disease compared with control

	Total compound	Hits	Statistic Q	Raw *p*	FDR adjusted *p*
Pyrimidine metabolism	39	2	41.42	<0.001	<0.001
beta-Alanine metabolism	21	2	22.32	<0.001	<0.001
D-Glutamine and D-glutamate metabolism	6	3	21.16	<0.001	<0.001
Pantothenate and CoA biosynthesis	19	5	14.23	<0.001	<0.001
Arginine biosynthesis	14	5	13.55	<0.001	<0.001
Nitrogen metabolism	6	2	27.86	<0.001	<0.001
Glycerolipid metabolism	16	2	20.82	<0.001	<0.001
Purine metabolism	65	3	15.84	<0.001	<0.001
Glycerophospholipid metabolism	36	1	25.60	<0.001	<0.001
Cysteine and methionine metabolism	33	4	13.58	<0.001	0.001
Glyoxylate and dicarboxylate metabolism	32	5	11.63	<0.001	0.001
Alanine, aspartate and glutamate metabolism	28	7	9.66	<0.001	0.001
Butanoate metabolism	15	2	12.27	0.001	0.01
Glutathione metabolism	28	4	10.62	0.002	0.01
Pentose and glucuronate interconversions	18	1	14.32	0.01	0.02
Arginine and proline metabolism	38	3	8.13	0.01	0.03
Porphyrin and chlorophyll metabolism	30	2	8.67	0.02	0.04
Taurine and hypotaurine metabolism	8	1	11.31	0.02	0.04
Thiamine metabolism	7	1	11.31	0.02	0.04
Histidine metabolism	16	2	8.76	0.02	0.04
Aminoacyl-tRNA biosynthesis	48	12	6.90	0.02	0.04
Galactose metabolism	27	3	7.26	0.03	0.04
Fatty acid elongation	38	1	8.99	0.03	0.07
Fatty acid degradation	39	1	8.99	0.03	0.07
Ascorbate and aldarate metabolism	8	2	7.21	0.04	0.07
Inositol phosphate metabolism	30	2	7.21	0.04	0.07
Valine, leucine and isoleucine degradation	40	4	6.43	0.04	0.07
Valine, leucine and isoleucine biosynthesis	8	4	6.43	0.04	0.07

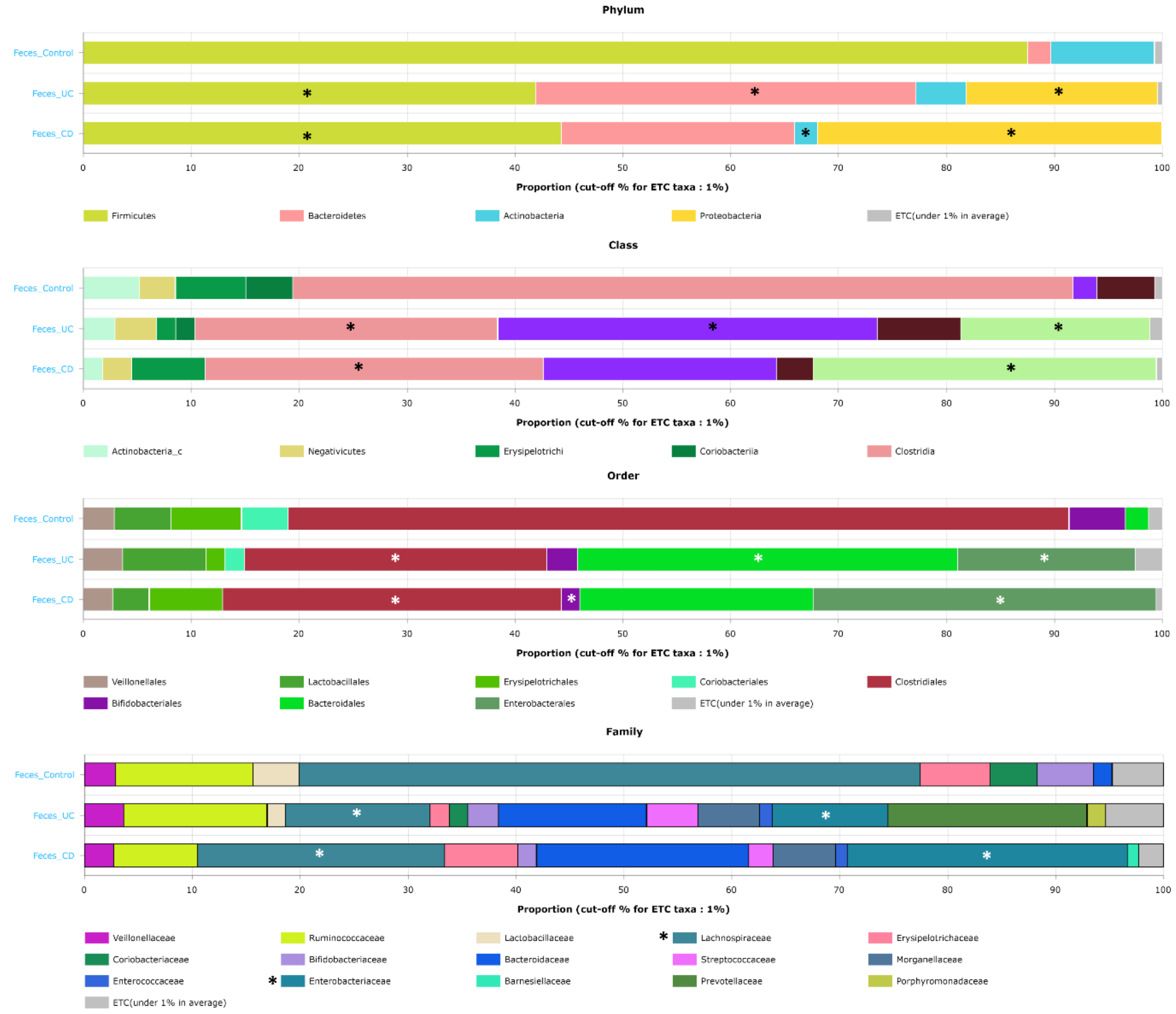

Supplementary Figure S1. Stacked bar chart of microbial composition of feces.

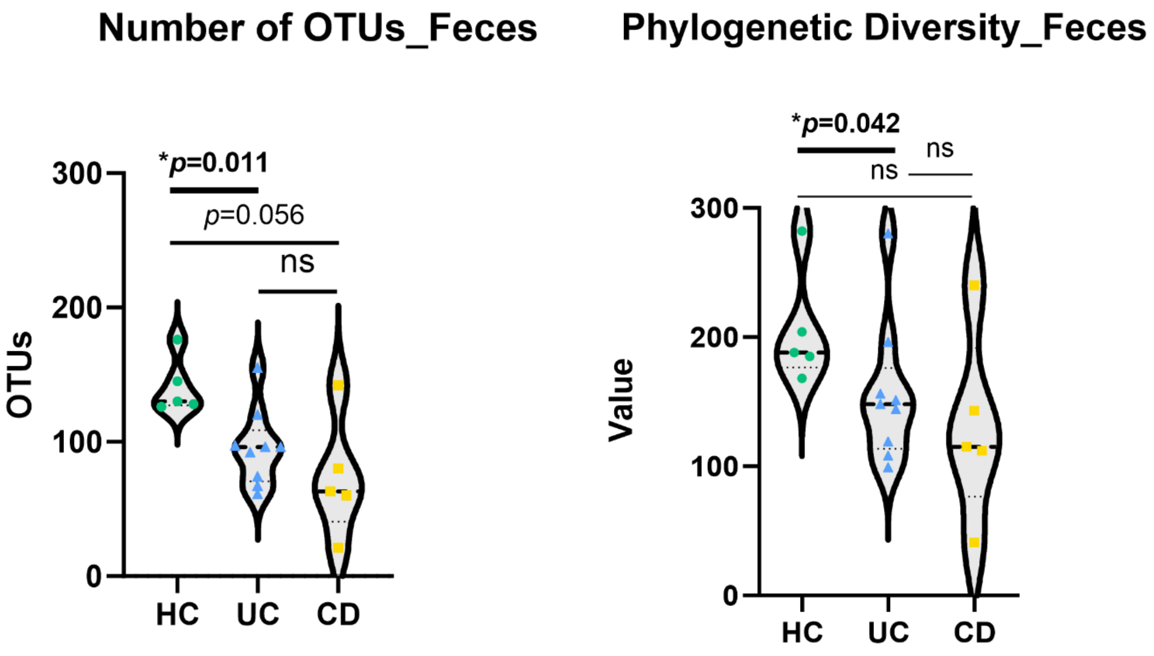

Supplementary Figure S2. Microbial α-diversity index from fecal samples.

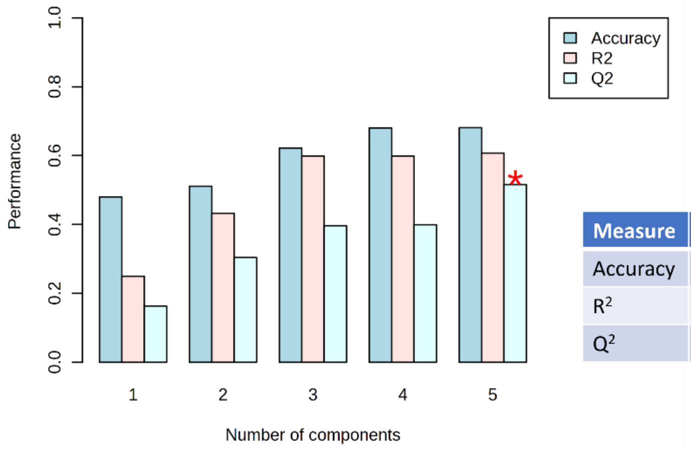

Measure	1 comps	2 comps	3 comps	4 comps	5 comps
Accuracy	0.48	0.51	0.62	0.68	0.68
R^2	0.25	0.43	0.60	0.60	0.61
Q^2	0.16	0.30	0.40	0.40	0.52

Supplementary Figure S3. Partial least squares discriminant analysis (PLS-DA) cross-validation of plasma metabolites by GC-TOF-MS analysis.

Cross-validation showed cumulative values of $R^2 = 0.61$ and $Q^2 = 0.52$ where R^2 indicates the variation shown by all 5 components in the model.

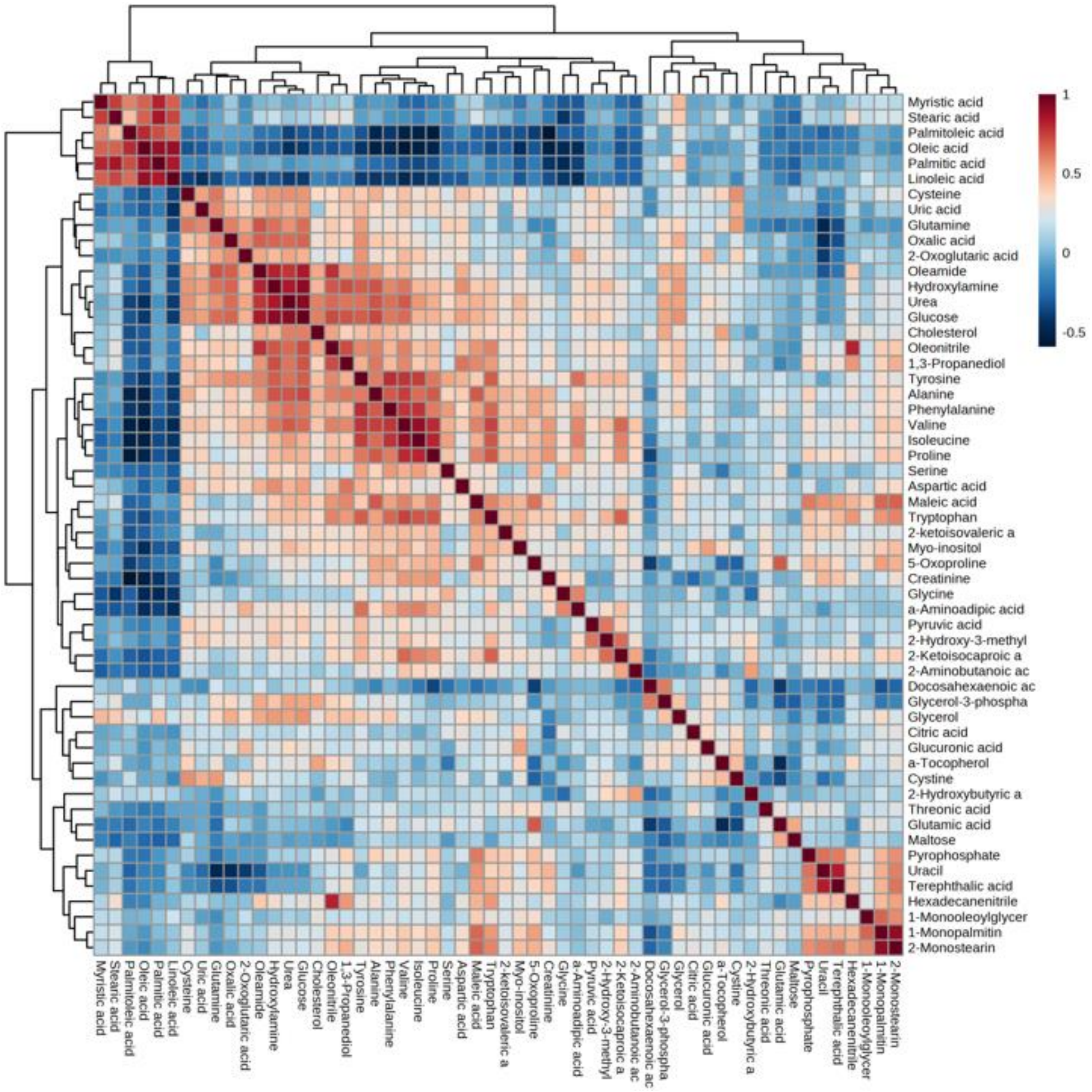

Supplementary Figure S4. Correlation analysis between metabolites.

Correlation heatmap by Spearman rank correlation analysis is shown. Metabolites within the same categories, such as fatty acids or amino acids, exhibited a positive correlation within each category.

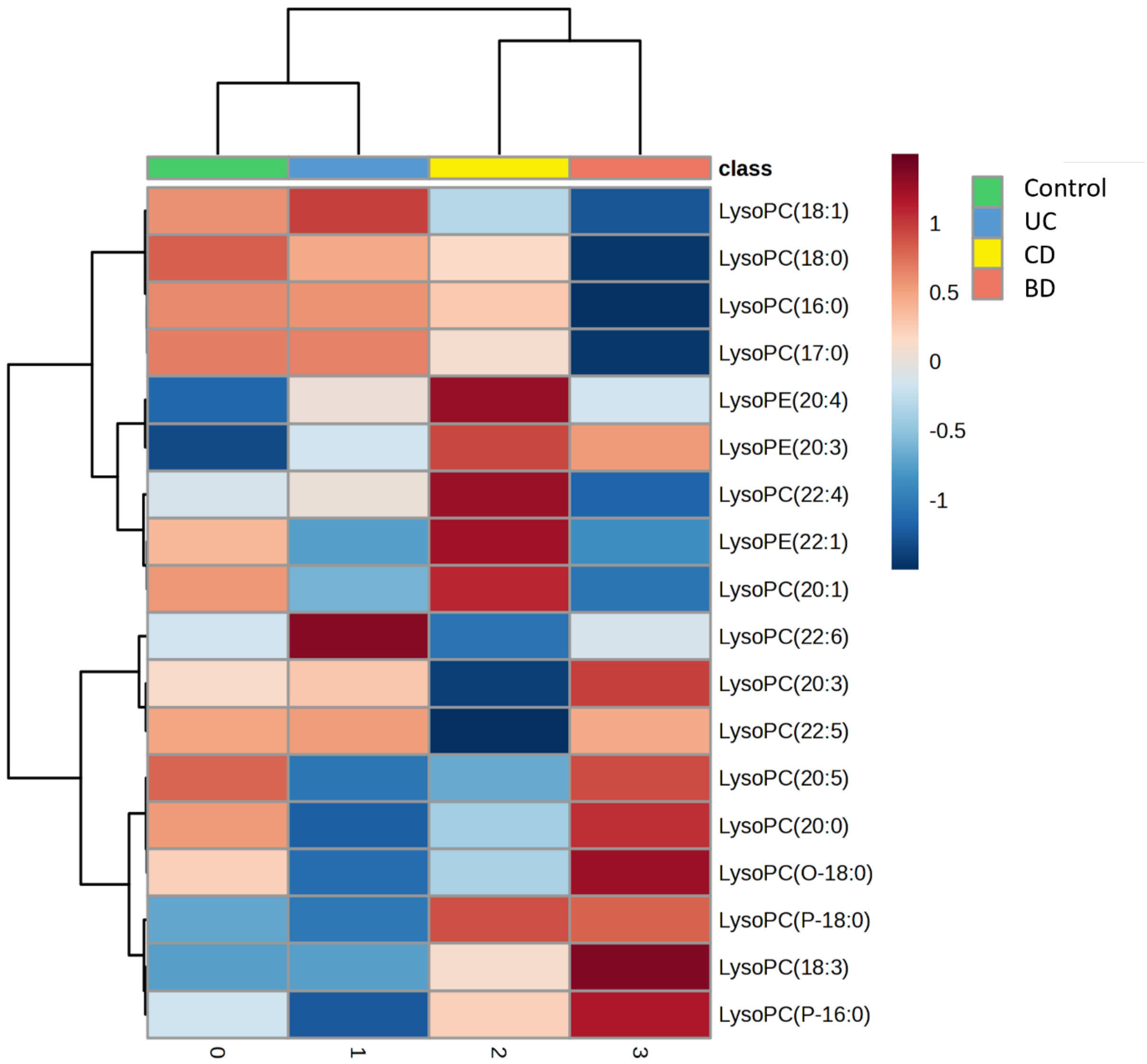

Supplementary Figure S5. Heatmap of plasma metabolite profiles using UPLC-Q-TOF-MS analysis by groups. UPLC-Q-TOF-MS: ultra-performance liquid chromatography–quadrupole/time-of-flight mass spectrometry, UC: ulcerative colitis, CD: Crohn's disease, BD: Behcet's disease
